# Designed to Heat and React: Fast Microwave-Engineered Iron Oxide Nanoflowers with Controlled Anisotropy for Magnetic Induction-Assisted Oxidation Processes

**DOI:** 10.1021/acsanm.6c01066

**Published:** 2026-05-08

**Authors:** Rafael Herrera-Aquino, Nahuel Nuñez, Raúl Magro, Sabino Veintemillas-Verdaguer, Elin L. Winkler, Ana Espinosa, María del Puerto Morales, Alvaro Gallo-Cordova

**Affiliations:** † Instituto de Ciencia de Materiales de Madrid, ICMM/CSIC, C/Sor Juana Inés de la Cruz 3, 28049 Madrid, Spain; ‡ Departamento Magnetismo y Materiales Magnéticos, Gerencia de Física, Centro Atómico Bariloche, Av. Bustillo 9500, 8400 San Carlos de Bariloche, Río Negro, Argentina; § Instituto de Nanociencia y Nanotecnología (CNEA-CONICET), Nodo Bariloche, Av. Bustillo 9500, 8400 San Carlos de Bariloche, Río Negro, Argentina; ∥ Instituto Balseiro, CNEA-UNCuyo, Av. Bustillo 9500, 8400 San Carlos de Bariloche, Río Negro Argentina

**Keywords:** iron oxide nanoflowers, microwave synthesis, magnetic hyperthermia, polyol method, reactive oxygen species

## Abstract

Developing synthetic routes that are both scalable and structurally controlled is critical for translating functional nanomaterials into real-world technologies. Here, we demonstrate the microwave-assisted polyol synthesis of iron oxide nanoflowers (NFs), reducing reaction times from 16 (autoclave) to only 120 min while maintaining >90% reproducibility. Crystallographic alignment of the primary cores is achieved through a carefully optimized heating profile: slow ramp (0.2 °C s^–1^) to 220 °C with a 20 min dwell, followed by microwave thermal sintering at 250 °C for 60 min to further enhance mesocrystal order. Magnetic anisotropy is tuned *in situ* by both sintering and cobalt incorporation, yielding improved magnetic performance and enhanced catalytic activity. electron paramagnetic resonance spectroscopy confirms reactive oxygen species generation with hydroperoxyl radicals (^•^OOH) as the dominant species, while synchrotron X-ray absorption spectroscopy reveals changes in electronic state and local order that, together with the presence of specific redox-active sites, collectively enhance catalytic activity. The most ordered samples (NF5, NF7) display the highest ROS yields and benchmark magnetic hyperthermia performance, with specific absorption rate values up to 710 W g_NPs_^–1^ (NF5) and 542 W g_NPs_^–1^ (NF7) at 200 kHz/24 kA m^–1^. Microwave-synthesized nanoflowers thus emerge as sustainable, reproducible, and multifunctional platforms coupling ROS generation with efficient magnetic heating.

## Graphical abstract

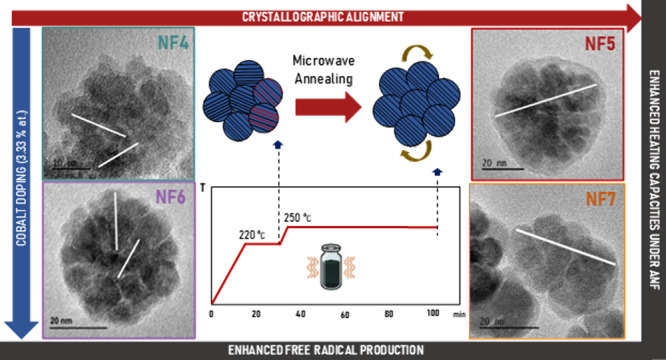

## 1 Introduction

Iron oxide nanoparticles have attracted significant attention due to their wide-ranging applications in biomedicine,^1,2^ environmental remediation,^3,4^ and catalysis.^5,6^ Among the synthetic strategies developed for their preparation, the polyol method has proven to be particularly versatile, enabling excellent control over nanoparticle size, morphology, crystallinity, and surface chemistry.^7,8^ Traditionally conducted in reflux systems or autoclaves, this method benefits from the high boiling point of polyols and their mild reducing and stabilizing properties.^9,10^ However, challenges remain in terms of reaction time, energy efficiency, and batch-to-batch reproducibility, especially when aiming for industrial scale.

In this context, microwave-assisted synthesis has emerged as a powerful alternative, offering fast and uniform heating, reduced reaction times, improved reproducibility,^11,12^ and scalability for continuous-flow processes.^13^ Unlike conventional heating, microwave irradiation couples directly with polar molecules and ions in the reaction medium through dipole polarization and ionic conduction mechanisms, leading to volumetric heating and more homogeneous nucleation conditions.^14^ It has been demonstrated that microwave-assisted solvothermal synthesis yields nanostructures with higher crystallinity compared to conventional methods because of accelerated reaction kinetics and uniform thermal profiles.^15,16^

These advantages are especially promising for the synthesis of multicore iron oxide nanostructures, such as nanoflowers, which exhibit superior properties in magnetic hyperthermia, magnetophoretic separation, and magnetic induction-assisted catalysis.^17–22^ These structures consist of crystallographically aligned primary units forming mesocrystals, whose collective magnetic behavior leads to enhanced anisotropy and responsiveness.^23^ Such oriented multicore assemblies can enter a superparamagnetic regime, yielding outstanding heating performance due to exchange coupling between aligned cores.^24,25^

Furthermore, a recent comprehensive study by Borchers et al. highlighted the dominant role of effective magnetic anisotropy, rather than morphology or composition, in determining the performance of iron oxide and cobalt ferrite nanoflowers in both magnetic particle imaging (MPI) and magnetic hyperthermia.^26^ They showed that subtle differences in particle structure, surface coating, or aggregation states had less influence than the anisotropy landscape in governing specific loss power (SLP) and point-spread function (PSF) outcomes. However, their work did not investigate or optimize the synthetic protocols that control such anisotropy during nanoparticle formation.

In this sense, and despite this potential, the direct and fast formation of mesocrystalline nanoflowers via microwave-assisted polyol synthesis remains largely unexplored. While several studies have combined microwave heating with polyol chemistry, these typically focus on single-core ferrite nanoparticles, and only a few address multicore structures. Among them, Sathya et al. reported a one-step microwave synthesis of Fe_3_O_4_ nanoclusters with tunable size and colloidal stability.^27^ However, their aggregates showed core disorder and lacked mesocrystalline coherence, resulting in suboptimal magnetic performance. Similarly, in a previous work, we also observed a morphological crossover from single-core Fe_3_O_4_ nanoparticles to multicore structures simply by switching the polyol from diethylene glycol to ethylene glycol; however, the resulting particles lacked internal ordering.^28^ Shaw et al., on the other hand, synthesized MnFe_2_O_4_/γ-Fe_2_O_3_ nanoflowers using a seeded microwave approach that enhanced heating via exchange coupling, but involved sequential growth steps and produced hybrid-phase structures without strict control over internal alignment.^29^ Considering the microwave-assisted coprecipitation method, Blanco-Andujar et al. developed iron oxide nanoflowers optimized for magnetic hyperthermia applications.^30^ They achieved moderate specific absorption rate (SAR) values (below 200 W g^–1^) that could be attributed to the lack of internal alignment within their structures, as mesocrystals are known to reach high SAR values (>700 W g^–1^).^25,31^ In addition, a recent study has determined small multicore structures as intermediates in the formation of cubic nanoparticles using octadecene as the reaction medium and with SAR values below 400 W g^–1^.^32^ Notably, none of these approaches has achieved the direct and sustainable, single-step formation of single-phase, structurally coherent mesocrystalline nanoflowers with tunable magnetic anisotropy, which remains critical for reaching high heating capabilities under an alternating magnetic field (AMF).

Mesocrystal formation typically proceeds via nonclassical crystallization pathways such as oriented attachment, rather than classical nucleation and growth.^33,34^ In previous work, we elucidated the formation mechanism of iron oxide nanoflowers using NMDEA-based polyol systems under autoclave conditions.^31^ We identified a crystallization crossover governed by solvent coordination, which allowed for the deliberate synthesis of either mesocrystals or single-core crystals. It seems that ligand solubility in the solvent controls the adsorption–desorption rate from the nanoparticle surface, which is crucial in determining whether mesocrystals remain as a multicore structure or fuse into single particles.^35^ In a subsequent study, we successfully scaled up this protocol to gram-scale production without sacrificing structural or magnetic quality, confirming the method’s robustness and relevance for applied technologies.^19^ Nevertheless, this system still presents some drawbacks, primarily related to high energy consumption from long reaction times and significant thermal gradients within this solvothermal procedure.

These mesocrystalline nanoflowers not only enhance magnetic performance but also show remarkable catalytic potential in oxidative environments through the generation of reactive oxygen species (ROS). Recent studies have demonstrated that the unique multicore architecture of iron oxide mesocrystals, characterized by large surface areas, surface defects, and collective magnetic effects, can significantly improve their ability to produce hydroxyl and superoxide radicals.^36^ For example, Fu et al. showed that oriented nanoflowers outperformed their non-oriented particles (nanoclusters) in terms of their peroxidase-like activity.^37^ Such behavior is particularly relevant for advanced oxidation processes (AOPs), where catalytic degradation of organic contaminants relies on efficient radical formation. In this context, the ability to synthetically control the degree of sintering and alignment within nanoflowers becomes essential not only for optimizing magnetic anisotropy but also for tuning surface redox activity in catalytic applications.

In the present study, we successfully translate the autoclave-based synthesis to a microwave-assisted platform, enabling faster and highly controllable fabrication of iron oxide nanoflowers while preserving their multicore mesocrystalline nature. We systematically tune the heating ramp, reaction time, and sintering steps to modulate the degree of core alignment and magnetic anisotropy. Additionally, we explore the incorporation of cobalt as a strategy to further tailor the magnetic properties. This work not only deepens our understanding of how microwave conditions influence mesocrystal formation but also offers practical insights into the scalable fabrication of multicore magnetic superstructures for hyperthermia and other field-responsive applications. By optimizing structural coherence and magnetic anisotropy, our approach aims to deliver materials with superior performance in both magnetic heating and free-radical generation, maximizing their potential for biomedical and catalytic technologies.

## 2 Results and Discussion

### 2.1 Synthesis of Iron Oxide Nanoflowers

The design of reproducible, scalable, and structurally precise synthetic routes is essential for obtaining magnetic nanomaterials with predictable and optimized functionality. In this work, we systematically explore the microwave-assisted polyol synthesis of iron oxide nanoflowers (NFs), aiming to translate the well-established autoclave route into a rapid and energy-efficient process without compromising structural order. Particular emphasis is placed on understanding the nucleation and growth sequence leading to multicore assemblies, as this mesocrystalline organization governs the resulting magnetic anisotropy, hyperthermia efficiency, and catalytic activity. The following sections detail the synthesis optimization, structural characterization, and property–structure correlations that establish a framework for rationally engineering NFs with tailored functionality.

#### 2.1.1 Early-Stage Multicore Assembly

To gain initial insight into the structural evolution of the nanoflowers, a first synthetic trial was performed under controlled microwave conditions using a fast-heating ramp (4 °C s^–1^), a target temperature of 220 °C, and a reaction time of 10 min, with the resulting sample labeled NF1 (Table 1).

**Table 1:** Summary of Structural Parameters and Yield of Iron Oxide Nanoflowers

Sample	Heating ramp (°C s^–1^)	Reaction time (min)	Temperature (°C)	TEM size (nm)	Core size (nm)	Crystal size (nm)	yield (%)
NF1	4	10	220	19 ± 3	7 ± 2	14	10
NF2	0.2	10	220	37 ± 6	8 ± 2	15	66
NF3	0.03	10	220	42 ± 3	9 ± 2	15	67
NF4	0.2	20	220	38 ± 5	11 ± 2	16	53
NF5	0.1–0.2	20 + 60	220 + 250	37 ± 4	21 ± 6	31	84
NF6	0.2	20	220	39 ± 9	8 ± 4	14	53
NF7	0.1–0.2	20 + 60	220 + 250	33 ± 4	16 ± 4	18	81

The transmission electron microscopy (TEM) micrograph (Figure 1a) revealed uniform multicore nanoparticles with an average size of 21 ± 3 nm (Figure 1b), composed of subunits/cores of approximately 6 ± 2 nm. X-ray diffraction analysis (Figure 1c) confirmed the presence of an inverse spinel-type iron oxide phase compatible with magnetite or maghemite, with a mean crystallite size of ∼12 nm, notably smaller than the overall particle diameter but greater than the TEM size of the cores. This discrepancy indicates that, although some degree of internal sintering has occurred, the primary building blocks have not fully coalesced into a single crystal and retain partial structural individuality.

**Figure 1:**
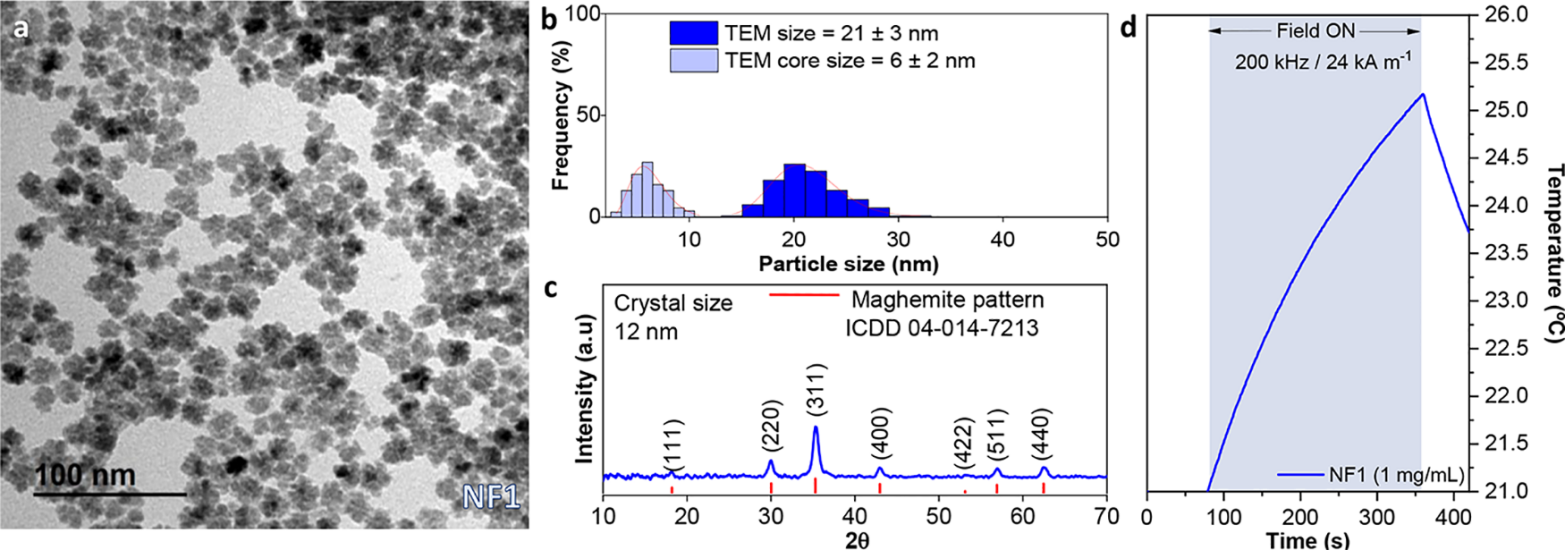
Structural characteristics and magnetic heating profile of the NF1 sample synthesized under microwave-assisted polyol conditions (220 °C, 10 min, 4 °C s^–1^ heating ramp). (a) TEM image showing uniform multicore nanoflowers with spherical morphology and well-defined aggregates. (b) Size distribution histogram derived from TEM analysis, showing a mean nanoflower diameter of 21 ± 3 nm and an average core size of 6 ± 2 nm. (c) XRD pattern with an estimated crystallite size of 12 nm. (d) Magnetic heating profile of NF1 (1 mg mL^–1^ in water) under an alternating magnetic field (200 kHz, 24 kA m^–1^), showing limited thermal response due to low internal anisotropy.

These findings align with previous studies reporting multicore iron oxide nanoflowers with partial internal coherence and modest functional properties. Similar structures in the 15–30 nm range have been shown to enable magnetic heating, although their SAR values typically remain limited due to incomplete crystallographic alignment.^30,38^ In this context, the NF1 sample in our study, while displaying the characteristic morphology of early-stage mesocrystals, still exhibits a relatively low synthetic yield, reaching only ∼10%. This is substantially lower than the ∼36% yield achieved in our previous work for ∼40 nm nanoflowers synthesized under autoclave conditions.^31^ In that system, the larger nanostructures were identified as late-stage intermediates in a nonclassical crystallization pathway, where multicore assemblies undergo progressive sintering and alignment to ultimately form single-core mesocrystals. Sample NF1, by contrast, represents an early aggregation state: small iron oxide nuclei are formed and loosely packed, but internal orientation and crystalline fusion are still incomplete. While such structures can support magnetic hyperthermia, their limited anisotropy and partial coherence restrict their heating efficiency (Figure 1d), with a SAR value of 80 W g^–1^, estimated by considering the slope from the heating profile at the first 30 s of the applied field. This, together with the low synthetic yield, underscores the need to optimize microwave reaction parameters, particularly reaction ramp, time, and postsynthetic sintering steps, to drive mesocrystal size and maturation, and access high-performing materials for both thermal and catalytic applications.

#### 2.1.2 Growth Modulation and Core Alignment

To investigate the influence of heating kinetics on nanoparticle growth, a series of syntheses were carried out using reduced heating ramps while maintaining a constant temperature (220 °C) and reaction time (10 min). As shown in Figure 2 (effect of the heating ramp), samples NF2 and NF3 were obtained using slow heating ramps of 0.2 and 0.03 °C s^–1^, respectively, and were compared to NF1, synthesized under a rapid heating ramp of 4 °C s^–1^.

**Figure 2:**
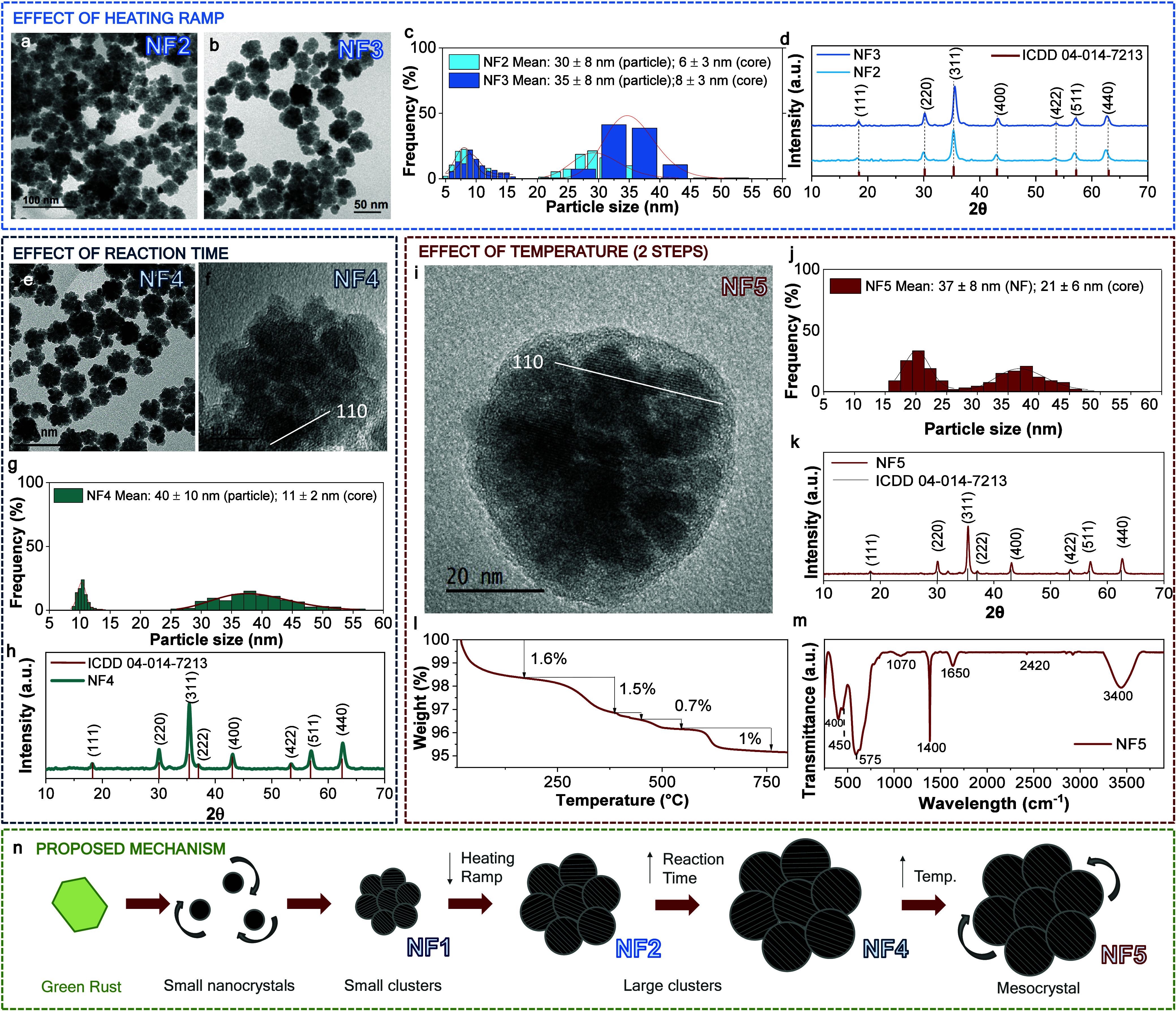
Structural evolution and mesocrystal formation of iron oxide nanoflowers synthesized via microwave-assisted polyol synthesis. Effect of heating ramp: (a, b) TEM images of NF2 and NF3 synthesized with different heating ramps (0.2 and 0.03 °C s^–1^), showing size increase and denser aggregates at slower ramping. (c) Particle and core size distributions. (d) XRD patterns indicate stability in crystallite size. Effect of reaction time: (e, f) TEM and HRTEM images of NF4 synthesized at a longer reaction time (120 min). (g) Particle and core size histograms. (h) XRD pattern of NF4. Effect of thermal post-treatment: (i) HRTEM image of NF5 after a secondary heating step (250 °C, 60 min), revealing enhanced crystallographic coherence. (j, k) Size distributions and XRD pattern of NF5. (l, m) TGA and FTIR curves of NF5. (n) Proposed mechanism: schematic representation of nanoflower growth through a nonclassical pathway involving green rust intermediates, formation of small nanocrystals, aggregation into clusters (disordered nanoflowers), and progressive alignment into mesocrystals upon thermal treatment.

TEM analysis (Figure 2a,b) reveals a clear increase in overall particle size with slower heating: NF2 and NF3 exhibit average diameters of 30 ± 8 and 35 ± 8 nm (Figure 2c), respectively, compared to 21 ± 3 nm in NF1. Despite this increase in size, the crystallite sizes determined by Scherrer analysis remain nearly unchanged between NF2 and NF3 (15.5 and 15.6 nm), indicating that the growth results mainly from enhanced aggregation and partial sintering of primary crystallites rather than individual crystal growth, preserving the inverse spinel-type magnetite or maghemite structure (Figure 2d). An intermediate sample synthesized at 0.05 °C s^–1^ (presented in Figure S1 of the Supporting Information) displays similar dimensions and a slightly lower crystallite size of 14.3 nm, reinforcing the notion that growth saturates beyond a certain heating rate threshold.

A slower heating ramp permits slower nucleation with greater interaction and aggregation of the initially formed nanocrystals, while structural densification through sintering and oriented attachment is preserved. However, once the aggregates reach a critical size, further decreases in heating rate do not result in additional particle growth. These trends are consistent with prior reports showing that heating rate plays a central role in balancing nucleation and growth dynamics in iron oxide systems.^39–41^ Based on these observations, the 0.2 °C s^–1^ ramp (NF2) was selected as the most efficient condition for subsequent experiments, offering reproducible growth, reduced energy input, and high yield (66%) among all conditions tested (Table 1).

On the other hand, Figure 2e–h examines the effect of reaction time on the structural evolution of iron oxide nanoflowers under optimized microwave conditions. The NF4 sample was synthesized at 220 °C using the optimal heating ramp of 0.2 °C s^–1^, extending the reaction time from 10 min (NF2, NF3) to 20 min (NF4). Additional experiments extending the reaction up to 120 min are included in Figure S2 of the Supporting Information. Structural analysis confirmed the spinel structure (Figure 2h), the crystallite size (≈15.8 nm), and the overall particle diameter, which reaches a plateau after 20 min, attributed to the depletion of available precursors at this stage, which limits further crystal growth. Thus, NF4 was selected as the optimally grown sample under this first heating stage.

At this point, the nanoflowers attain sizes (40 ± 10 nm, Figure 2e,g) and morphologies similar to those obtained under autoclave conditions in our previous work,^31^ yet still exhibit no full crystallographic fusion. High resolution transmission electron microscopy (HRTEM) imaging of NF4 (Figure 2f) reveals multiple crystalline domains without coherent lattice continuity across the structure, indicating that internal alignment is incomplete. This contrasts with our previous solvothermal studies, where longer reaction times (∼24 h) led to full sintering into large single crystals as part of a nonclassical growth pathway.^33,34^ Under this specific microwave equipment, safety constraints limit the reaction duration at 220 °C to 120 min, which precludes reaching the degree of crystallographic integration achievable under autoclave conditions.

To overcome this limitation, Figure 2i–m explores a second thermal step (see microwave temperature profile in Figure S3 in the Supporting Information) to enhance internal alignment. Following a 20 min growth at 220 °C (0.2 °C s^–1^ ramp), the temperature was increased to 250 °C using a 0.1 °C s^–1^ ramp rate, followed by a 60 min dwell (the maximum safe duration at this temperature for the microwave reactor). The resulting NF5 sample maintains an external diameter (37 ± 8 nm, Figure 2j) consistent with sample NF4, but exhibits a markedly larger crystallite size of 31 nm (Figure 2k), while the primary cores observed by TEM remain around 21 ± 6 nm (Figure 2j). This suggests that once the chemical process has concluded, the nanostructure of the particles evolves by sintering and partial coalescence, resulting in larger cores and coherently aligned crystalline domains. HRTEM analysis (Figure 2i) supports this interpretation, revealing well-defined lattice fringes extending across multiple cores, evidence of mesocrystal formation. Additional HRTEM and selected area electron diffraction (SAED) analysis (Figure S4) further support the structural evolution from disordered multicore assemblies to partially aligned nanoflowers. While NF4 exhibits a ring-like diffraction pattern characteristic of randomly oriented nanocrystals, NF5 shows the appearance of discrete spots superimposed on rings, indicating enhanced crystallographic alignment consistent with mesocrystalline ordering. Detailed sintering analysis was performed with shorter isothermal dwells at the second heating step (5 and 30 min), and the samples exhibit clearly less-sintered phases due to lower crystal sizes of 23 and 27 nm, respectively (Figure S5, Supporting Information). A faint shell-like contrast is observed around the nanoflowers, attributed to a thin layer of residual diethylene glycol adsorbed on the surface, consistent with Fourier Transform Infrared Spectroscopy (FTIR) and thermal gravimetric analysis (TGA) results. This layer mainly contributes to colloidal stability and is not expected to affect the magnetic or catalytic properties.

Further colloidal and compositional characterization of the NF5 sample was performed. Specifically, dynamic light scattering measurements at neutral pH revealed a hydrodynamic diameter of approximately 70 nm in intensity, accompanied by a low polydispersity index (PDI = 0.1), as shown in Figure S6 of the Supporting Information. The ζ-potential profile indicated an isoelectric point around pH 7, consistent with the absence of significant surface functionalization beyond residual DEG molecules and trace nitrate species (Figure S7). TGA suggested that surface-bound DEG accounted for less than 3% of the total mass (Figure 2l). The FTIR spectrum (Figure 2m) further supported these findings. A band at 1380 cm^–1^ was attributed to nitrate species adsorbed during the acidic oxidation step, while the peak at 1070 cm^–1^ corresponds to C–O–C stretching vibrations characteristic of DEG molecules.^42^ A broad absorption around 3400 cm^–1^ arises from surface hydroxyl groups and physisorbed water. Additionally, the band near 1650 cm^–1^ is assigned to the bending mode of molecular water (H–O–H), and a weaker feature at ∼2420 cm^–1^ is consistent with adsorbed atmospheric CO_2_, commonly observed in hydroxylated surfaces.^43^ The characteristic Fe–O stretching vibrations of iron oxide phases appear in the range of 400–600 cm^–1^.^44^

Figure 2n presents a schematic summary of the formation mechanism and the structural evolution of iron oxide nanoflowers under microwave-assisted synthesis. It illustrates how each synthetic parameter (heating ramp, reaction time, and a secondary temperature step) modulates the aggregation, growth, and alignment of primary nanocrystals within the multicore architecture. Initially, rapid heating promotes the formation of precursor species, likely associated with green rust-like intermediates at the nanoparticle surface,^31^ which subsequently evolve into loosely assembled aggregates with limited internal coherence. As the ramp slows, nanocrystals interact more effectively, promoting partial sintering and structural densification. However, alignment remains incomplete at 220 °C, even after extended reaction times. Introducing a second thermal stage at 250 °C enables further sintering and promotes crystallographic alignment, as evidenced by increased crystallite size and improved HRTEM contrast, without inducing full core fusion.

This mechanistic overview highlights the critical interplay between kinetic control and thermal input in steering the transition from disordered polycrystalline clusters to mesocrystalline structures with tailored magnetic anisotropy. As supported by previous studies, slow heating ramps and multistep heating protocols facilitate structural densification, promoting oriented attachment and crystallographic alignment of primary nanocrystals. Though full fusion into single crystals, as observed under autoclave conditions, is not achieved, this controlled sintering is a key step toward achieving the magnetic anisotropy necessary for high-performance hyperthermia and catalytic applications, as it will be further analyzed in the following sections.

#### 2.1.3 Cobalt Incorporation

Figure 3a–c summarizes the effect of cobalt incorporation on the structural and morphological features of the iron oxide nanoflowers. The replacement of iron cations with the more anisotropic Co^2+^ cation allows increasing the magnetocrystalline anisotropy up to 20 times with respect to that of pristine magnetite while keeping the total magnetic moment almost unchanged.^45^ A nominal cobalt content of 3.33 atom % (2.53 wt %) was selected, corresponding to the stoichiometry Co_0.1_Fe_2.9_O_4_ (confirmed by ICP-OES elemental analysis), to ensure minimal metal doping while mitigating potential toxicity associated with higher cobalt concentrations.

**Figure 3:**
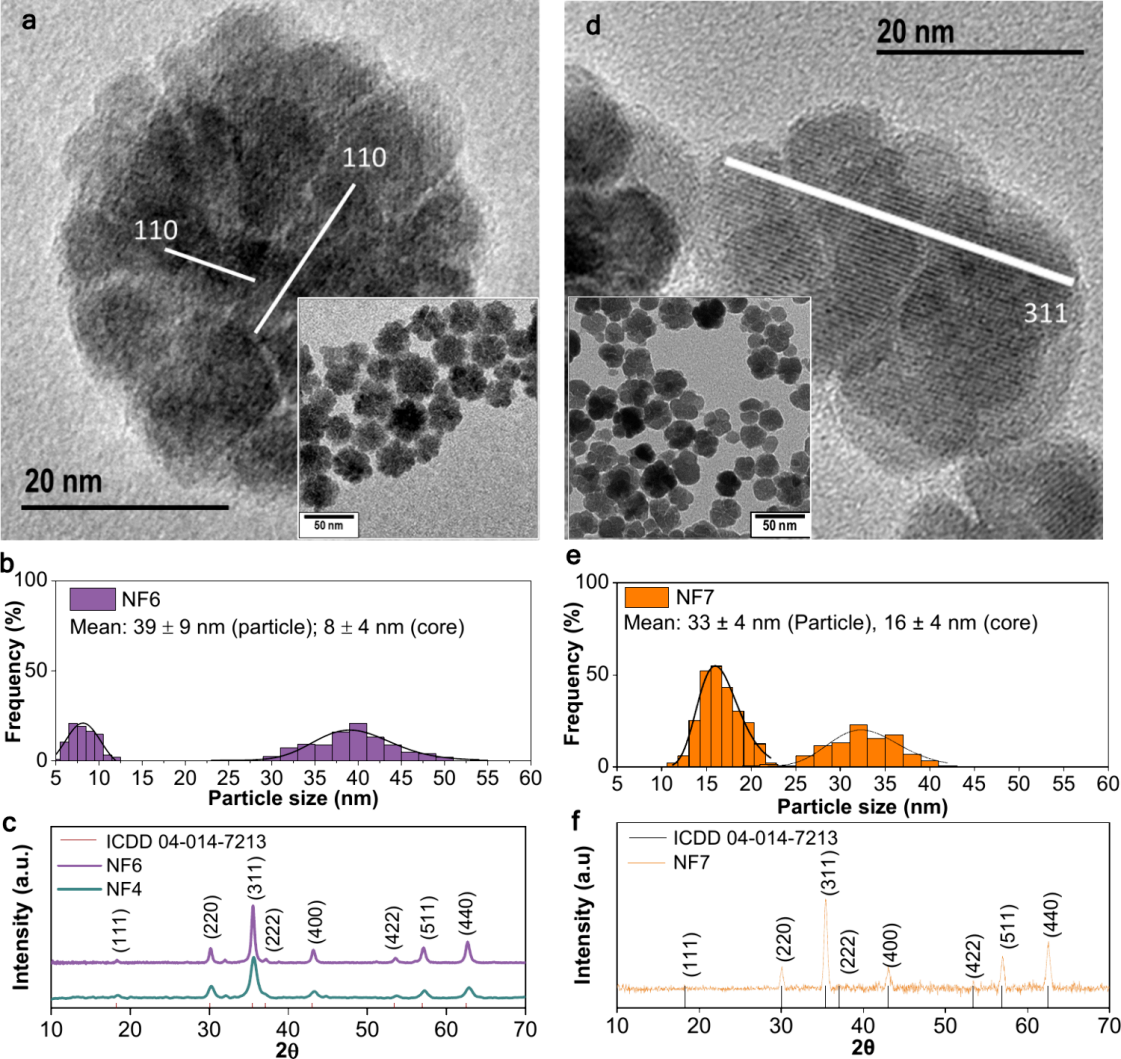
Structural and morphological characterization of cobalt-doped nanoflowers (on the left, NF6 non-sintered, and on the right, NF7 sintered). Effect of cobalt incorporation (3.33 atom %) on nanoflowers (NF6): (a) High-resolution TEM image showing a multicore structure with individual core lattice fringes, indicating local crystallinity without collective alignment. Inset: low-magnification TEM image. (b) Size distribution histogram of particle diameters and core sizes obtained from TEM analysis. (c) XRD patterns comparing NF6 and undoped NF4 nanoflowers, showing a shift toward lower 2θ angles indicative of lattice expansion upon cobalt incorporation. Effect of microwave thermal treatment (a secondary heating step, 250 °C, 60 min) on cobalt-doped nanoflowers (3.33 atom %, NF7): (d) High-resolution TEM image showing enhanced crystallographic alignment of cores after two-step heating (220 + 250 °C). Inset: corresponding low-magnification TEM image. (e) Size distribution histogram showing slight densification after thermal treatment. (f) XRD pattern of NF7 confirming improved crystallinity with sharper diffraction peaks compared to NF6.

The incorporation of cobalt results in nanoflowers (NF6) with an average particle size of 39 ± 9 nm and a core size of 8 ± 4 nm, similar to the undoped samples (Figure 3b). High-resolution TEM images (Figure 3a) reveal multicore architectures with visible lattice fringes corresponding to the (110) planes, indicating good crystallinity at the core level but without collective crystallographic alignment across the flower structure. Powder XRD analysis (Figure 3c) shows a slight shift toward lower 2θ values compared to the undoped NF4 sample, reflecting an expansion of the lattice parameter from 8.351 Å (NF4) to 8.387 Å (NF6). This increase is consistent with the substitution of Fe^3+^ ions (ionic radius 0.645 Å) by larger Co^2+^ ions (0.745 Å) within the spinel lattice.^45^

To further achieve a crystallographic arrangement of the Co-doped sample, a two-step microwave heating protocol was performed (NF7, Figure 3d–f). After initial nucleation at 220 °C for 20 min, the temperature was ramped up to 250 °C at 0.1 °C s^–1^ and held for 60 min. This thermal treatment promotes partial sintering and alignment of the cores, as observed in the high-resolution TEM images (Figure 3d), where continuous lattice fringes extend across multiple cores. Structurally, NF7 exhibits a slight densification with a reduced particle size of 33 ± 4 nm (Figure 3e). Importantly, the crystallite size further grows to 18 nm, supporting the enhanced crystallographic coherence achieved during the thermal step. The corresponding XRD pattern (Figure 3f) displays sharper and more intense diffraction peaks compared to NF6, confirming the improved crystallinity without phase decomposition. In this sense, and as in the undoped samples (NF5), it has been proved here that it is possible to finely modulate the internal architecture and magnetic anisotropy of the cobalt-doped iron oxide nanoflowers. As mentioned before, such control is crucial for optimizing their performance in magnetic hyperthermia and field-responsive catalytic applications, as will be discussed in the subsequent section. Table 1 summarizes the structural parameters of all samples (NF1–7), together with their yield. A remarkable result is the high yield obtained for the multistep heating samples, of 81 and 84% for NF5 and NF7. Typically, mesocrystals arise as transient intermediates with yields of around 40%.^19^ In this case, the controlled multistep heating allowed reaching an optimal sintering state, where the particles remain as aligned, semisintered multicore assemblies. This strategy maximizes their yield without driving the reaction toward the complete single-core transformation, where a 100% yield is obtained. Furthermore, the observed variations in yield among all samples are attributed to the interplay between nucleation and growth kinetics. Fast heating conditions lead to the formation of small, weakly aggregated structures with low yield, whereas slower ramps and extended thermal treatment promote particle growth, aggregation, and sintering into more stable nanoflowers, resulting in higher yields.

### 2.2 Magnetic Properties and Hyperthermia

Figure 4a,b presents the magnetic hysteresis loops (*M*–*H* curves) for NF4 and NF5 (sintered) nanoflowers recorded at 290 and 5 K. The extracted magnetic parameters, saturation magnetization (*M*_S_), remanent magnetization (*M*_R_), and coercive field (*H*_C_), are summarized in Table 2. At 290 K, NF5 exhibits a saturation magnetization of 89 Am^2^ kg^–1^, an *M*_R_/*M*_S_ ratio of 0.01, and a coercive field of 0.7 kA m^–1^, while NF4 displays a slightly lower *M*_S_ of 80 A m^2^ kg^–1^ but a higher *M*_R_/*M*_S_ ratio of 0.1 and *H*_C_ of 3.4 kA m^–1^. These low *M*_R_/*M*_S_ ratios and coercive fields at room temperature are indicative of thermally activated magnetization reversal and superparamagnetic-like behavior.

**Figure 4:**
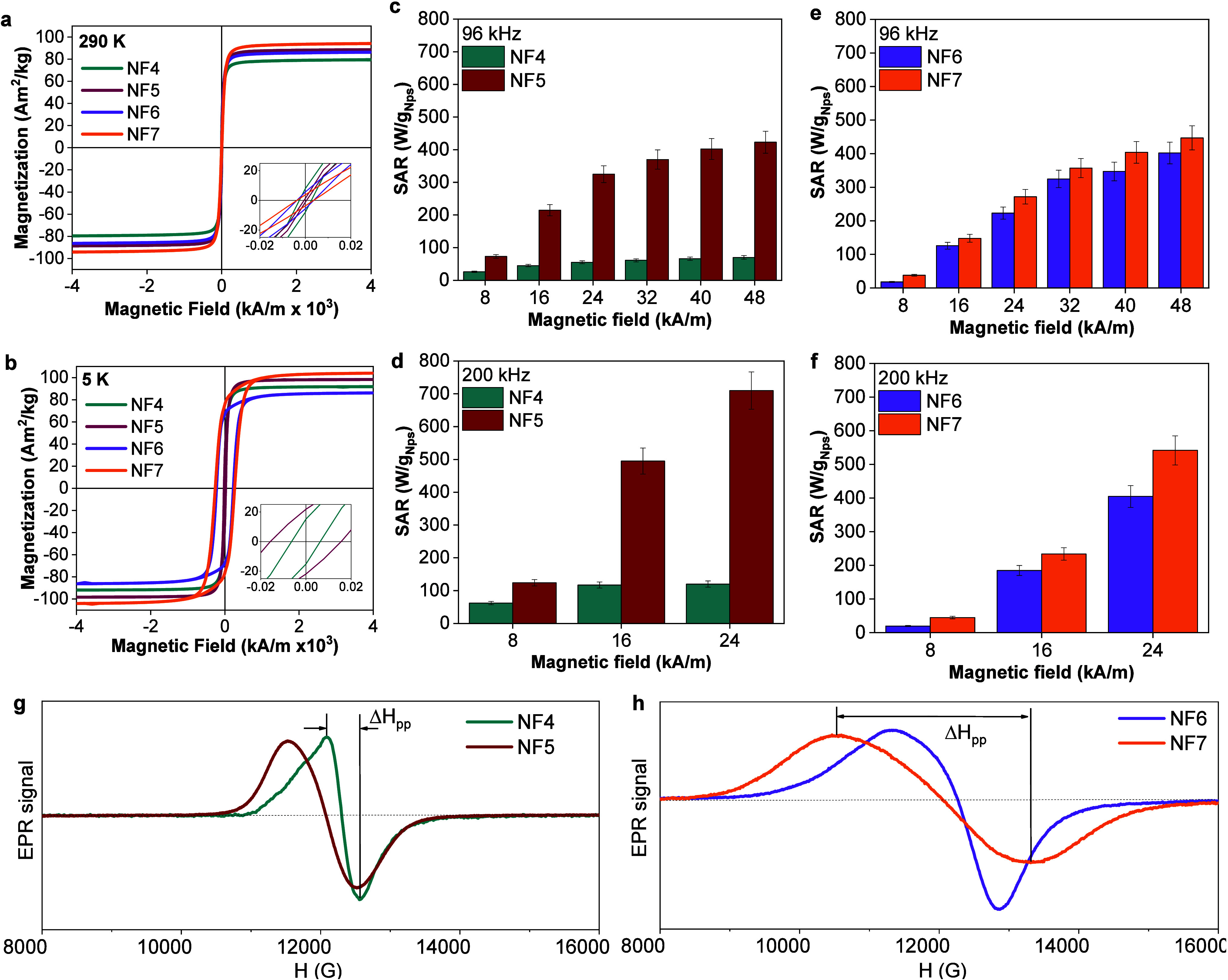
Magnetization curves of NF4–7 measured at (a) 290 K and (b) 5 K. Insets show enlarged views of the low-field region. Magnetic hyperthermia performance of NF4–7: SAR as a function of applied field at (c, e) 96 kHz and (d, f) 200 kHz. Ferromagnetic resonance spectra of (g) NF4 and NF5 and (h) NF6 and NF7, highlighting shifts in the resonance field (*H*_res_) associated with sintering and cobalt incorporation. NF4 and NF5 correspond to undoped samples (nonsintered and sintered, respectively), while NF6 and NF7 correspond to Co-doped samples (nonsintered and sintered, respectively).

**Table 2:** Magnetic Parameters of Iron Oxide Nanoflowers

	*M*_S_ (Am^2^ kg ^–^^1^)		*H*_C_ (kA m^–^^1^)		*M*_R_/*M*_S_		SAR (W g_NPs_^–1^)		*K*_eff_ (kJ m^–3^)
Sample	290 K	5 K	290 K	5 K	290 K	5 K	96 kHz	200 kHz	290 K
NF4	80	92	3.4	6.6	0.10	0.16	70	120	6.5
NF5	89	98	0.7	15.8	0.01	0.22	423	710	15
NF6	86	87	4	220	0.07	0.70	402	405	22
NF7	95	105	4	261	0.04	0.77	447	542	44

When cooling to 5 K, both systems transition into a blocked state with significantly enhanced coercivity and remanence: NF5 reaches 15.8 kA m^–1^ with an *M*_R_/*M*_S_ of 0.22, and NF4 reaches 6.6 kA m^–1^ with an *M*_R_/*M*_S_ of 0.17. The increase in *M*_R_/*M*_S_ ratios reflects reduced thermal agitation, although both are far from the expected value for single-domain particles (0.5). The *M*_R_/*M*_S_ values agree better with multidomain particles, which show lower squareness due to their complex magnetic domain structures and incoherent magnetization rotation.^46,47^ Interestingly, at low temperature, NF5, which initially exhibited a softer response at RT, shows higher coercivity compared to NF4. This inversion suggests that the enhanced structural coherence of NF5 promotes stronger intercore magnetic coupling as thermal agitation decreases. Such behavior is consistent with previous reports on multicore nanoparticles, where increased core alignment and exchange coupling lead to collective magnetic behavior and higher magnetic anisotropy.^21,31^ Thus, the observed trends are consistent with a system evolving from disordered, weakly interacting multicore clusters (NF4) toward more structurally coherent, magnetically coupled mesocrystals (NF5), leading to enhanced magnetic anisotropy.

When Co is introduced into the NF (sample NF6), the shape of the hysteresis loop at low temperature undergoes a pronounced modification, increasing the coercivity and the squareness (*M*_R_/*M*_S_), while *M*_S_ is not changed significantly, being close to the bulk value for magnetite or cobalt ferrite (90 Am^2^ kg^–1^ at 10 K).^48^ Furthermore, saturation magnetization, coercivity, and squareness slightly increased with the extra heating step (NF7). In all cases, saturation magnetization values are among the highest that can be found in the literature for similar substoichiometric Co ferrite particles larger than 10 nm and obtained at *T* > 200 °C.^49^

For both samples, NF6 and NF7, symmetry changes of the magnetic anisotropy from uniaxial to cubic concur, leading to high *H*_C_ values, i.e., 220–261 kA m^–1^ at low temperature, in addition to an increase in *M*_R_/*M*_S_ up to 0.7–0.77, respectively. Similar coercivities and squareness values at low temperature have been reported for substoichiometric cobalt-ferrite NPs with a size larger than ∼35 nm, showing an incoherent magnetization reversal process (curling).^50,51^ However, in those cases, the squareness at RT was close to 0.5, indicating that the system remains magnetically blocked and does not exhibit the superparamagnetic behavior expected for smaller particles, thereby hindering applications requiring reversible magnetization. In contrast, our particles display superparamagnetic behavior at RT even for comparatively large particles (∼40 nm), which represents a clear advantage for combining high magnetic moment with reversible magnetization at room temperature. This highlights the effectiveness of this strategy, introducing cobalt to enhance magnetic anisotropy in the NF, leading to a superparamagnetic system at room temperature that minimizes magnetic interactions, improving colloidal stability.

SAR measurements were carried out through field-sweep experiments at two frequencies, 96 and 200 kHz (Figure 4c–f and Table 2). As expected, SAR increased with the applied field in all cases. The maximum values obtained for each sample at 96 kHz followed the order NF7 (447 W g_NPs_^–1^) > NF5 (423 W g_NPs_^–1^) > NF6 (402 W g_NPs_^–1^) > NF4 (70 W g_NPs_^–1^), while at 200 kHz the trend shifted to NF5 (710 W g_NPs_^–1^) > NF7 (542 W g_NPs_^–1^) > NF6 (405 W g_NPs_^–1^) > NF4 (120 W g_NPs_^–1^). The uncertainty in SAR values was estimated to be approximately ±5–10% (see Figure 4, mainly arising from the determination of the initial slope and experimental variability.

The obtained SAR values are in good agreement with those previously reported for analogous nanoflowers synthesized via autoclave methods, further confirming the successful translation of the synthesis to a microwave-assisted approach.^31^ In addition, these results are consistent with the enhanced heating performance expected for structurally coherent nanoflowers, as discussed in the Section 1.

At 96 kHz, cobalt-doped samples (NF6 and NF7) tend to outperform their undoped counterparts (NF4 and NF5). This behavior is consistent with the increased magnetic anisotropy introduced by cobalt, which enhances the heating efficiency at lower frequencies. However, considering the experimental uncertainty in SAR values, these differences should be interpreted with some caution. Similar trends have been reported for Co-substituted ferrites, where doping can optimize SAR.^52^ At 200 kHz, however, the trend reverses: the sintered, crystallographically aligned NF5 outperforms even the Co-doped NF7. In this frequency regime, structural coherence becomes the dominant factor. Alignment across primary cores reduces spin disorder and allows for more efficient collective magnetization dynamics, resulting in superior heating efficiency. This is consistent with reports showing that mesocrystalline iron oxide assemblies benefit from enhanced cooperative effects that maximize the SAR under high-frequency fields.^53^ It should also be noted that cobalt ferrites typically require higher applied fields to fully open their hysteresis loops, and thus, the maximum heating potential of Co-doped nanoflowers may not be fully realized under the AMF conditions used here.^54^

To gain further insight into the dynamic magnetic behavior of the nanoflowers, room-temperature ferromagnetic resonance (FMR) measurements were carried out on randomly oriented samples using an excitation frequency of 39.9 GHz (Q-band). The FMR spectra of cobalt-free nanoflowers (NF4 and NF5) and cobalt-doped nanoflowers (NF6 and NF7) are presented in Figure 4g,h, respectively. All the samples exhibit asymmetric resonance lines, a characteristic powder-pattern line shape that arises from the random distribution of the anisotropy axis of the magnetic nanoparticles relative to the external magnetic field. It is well established that the magnetic anisotropy field can be directly obtained from the peak-to-peak line width, Δ*H*_p‑p_, defined as the separation between the most widely separated peaks in the spectra as signaled in Figure 4.^55^ NF4 displays a resonance line with relatively small linewitdh, where the additional heating step (NF5) and the cobalt incorporation (NF6 and NF7) lead to increasing asymmetric line shapes and broadening of Δ*H*_p‑p_. For randomly oriented nanoparticles with uniaxial symmetry, the anisotropy field *H*_K_ is related to the FMR peak-to-peak line width (Δ*H*_p‑p_), which reflects the distribution of internal effective fields associated with magnetic anisotropy, through the expression *H*_K_ = 2/3Δ*H*_p‑p_. The factor 2/3 arises from the angular averaging of the anisotropy axes with respect to the applied field. The anisotropy field is related to the effective anisotropy constant through *H*_K_ = 2*K*_eff_/*M*_S_. The estimated values of *K*_eff_ for the NF4–7 systems obtained from the line width are summarized in Table 2. Although cobalt ferrite exhibits cubic magnetocrystalline anisotropy in bulk, the effective anisotropy in nanoparticle systems is often dominated by surface, shape, and interaction contributions, leading to an overall uniaxial-like behavior. Therefore, the use of this approach provides an effective description of the magnetic anisotropy in the present system.

The effective anisotropy constant results from the contribution of several terms, including magnetocrystalline (*K*_mc_), shape (*K*_sh_), surface (*k*_sur_), etc. Interestingly, NF4 exhibits a *K*_eff_ of 6.5 kJ m^–3^, comparable to the bulk magnetocrystalline anisotropy value reported for maghemite (e.g., *K*_mc_ ≈ 5 kJ m^–3^),^56^ despite the small core size of 11 nm. This suggests that the intercore interaction induces magnetic frustration of the surface spins, thereby reducing the effect of the surface anisotropy. In contrast, NF5, which underwent the additional heating step during synthesis, shows a considerably larger *K*_eff_ of 15 kJ m^–3^. This enhancement is likely related to the sintering process revealed by structural characterization, where partial merging of the cores promotes preferential crystallographic alignment, thereby reinforcing the overall anisotropy of the nanoflowers.

Upon cobalt incorporation, *K*_eff_ increases markedly, from 6.5 kJ m^–3^ in NF4 to 22 kJ m^–3^ in NF6, consistent with the expected enhancement of the magnetocrystalline anisotropy constant in cobalt-doped spinel ferrite, which can reach values of ∼180–300 kJ m^–3^ in stoichiometric cobalt ferrite (CoFe_2_O_4_).^57^ Thus, even the relatively small cobalt content in NF6 (Co_0.1_Fe_2.9_O_4_) accounts for the observed increase. Notably, sample NF7, which combines cobalt doping with the extra heating step, displays the highest anisotropy constant of 44 kJ m^–3^. Interestingly, this increase exceeds the sum of the individual enhancements observed for NF5 (extra heating) and NF6 (cobalt doping), suggesting a synergistic effect between both strategies in boosting anisotropy.

These results demonstrate that both directional recrystallization induced by sintering and controlled cobalt doping provide effective routes to tuning the anisotropy constant of ferrite nanoflowers. Such control over the magnetic anisotropy is essential for optimizing their performance in magnetic hyperthermia applications.

### 2.3 Reproducibility of the Microwave-Assisted Synthesis

The reproducibility of the optimized microwave protocol was evaluated by preparing seven independent batches of NF4 nanoflowers under identical reaction conditions. Figure 5 summarizes the consistency of key physicochemical and magnetic properties, including yield, TEM particle and core sizes, XRD crystal size, DLS hydrodynamic size, saturation magnetization (*M*_S_), coercivity (*H*_C_), magnetic susceptibility (χ), and SAR under an AMF of 200 kHz and 24 kA m^–1^. The reproducibility values were estimated as (1 – σ/*x̅*) × 100, where σ is the standard deviation and *x̅* is the mean value. Figures S8–S12 of the Supporting Information include the detailed characterization of all NF4 samples, while Table S1 summarizes their structural, colloidal, and magnetic parameters.

**Figure 5:**
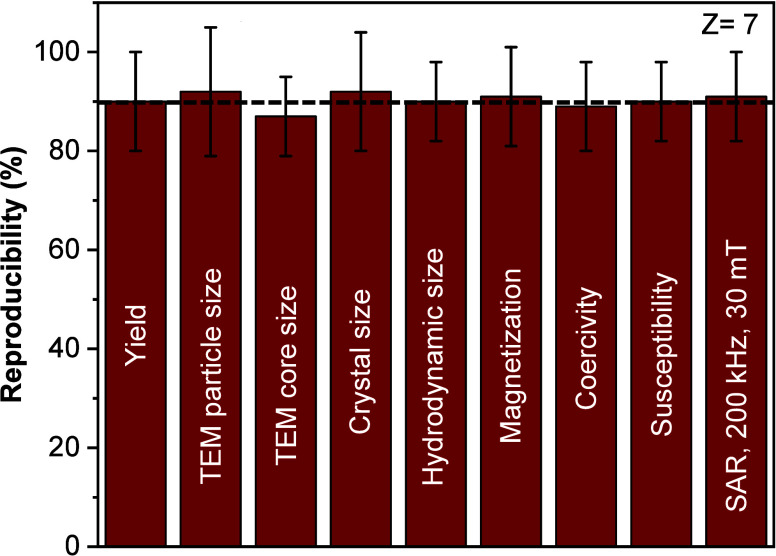
Reproducibility assessment of the microwave-assisted synthesis protocol for NF4 (undoped and nonsintered) based on 7 independent batches. The bar graph shows the mean values and standard deviations for key physicochemical and magnetic properties, including reaction yield, TEM particle size, core size, crystallite size, hydrodynamic size, saturation magnetization (*M*_S_), coercivity (*H*_C_), magnetic susceptibility (χ), and specific absorption rate (SAR) under 200 kHz and 24 kA m^–1^ conditions. The low standard deviations observed across all parameters indicate excellent batch-to-batch reproducibility, validating the robustness of the synthetic protocol for scalable and reliable nanoparticle production.

The obtained reproducibility values for most properties remained above 90%, demonstrating excellent batch-to-batch consistency. In particular, the particle size, core size, and crystallite size exhibited minimal variation, indicating robust control over the nucleation and growth processes. Similarly, magnetic properties such as *M*_S_, *H*_C_, and χ displayed low dispersion, reflecting the high degree of structural and magnetic homogeneity achieved under the microwave-assisted conditions.

Of special interest is the SAR reproducibility of 91%, which is crucial for practical applications in magnetic induction-assisted processes, where consistent heating efficiency is required. The changes in yield can be attributed to minor variations in precursor mixing, washing of the product, and sampling during parallel synthesis.

The automated multiple-batch production was the chosen strategy for scaling the nanomaterial production. It allows obtaining 60 mg per batch in 20 min, corresponding to around 360 mg h^–1^. In addition, the short reaction times achieved under microwave heating make it possible to integrate this method into a continuous-flow system. The use of a continuous flow system has been shown to provide significantly higher production, reaching several grams per hour. Beyond productivity, continuous flow synthesis also offers advantages in terms of reproducibility, precise control of reaction parameters, and easier integration into industrial processes, which makes it a more suitable strategy for large-scale nanomaterial manufacturing.^13^ Because of the reduction of reaction times, our microwave-assisted procedure is an excellent candidate for continuous flow production. Nevertheless, some extra considerations should be made before proceeding to this step.

### 2.4 Catalytic Effect for Free Radical Generation

The Fenton-like catalytic activity of the NFs was investigated by electron paramagnetic resonance (EPR), focusing on the generation of hydroperoxyl (^•^OOH) and hydroxyl (^•^OH) radicals in the presence of H_2_O_2_ (Figure 6). The EPR signal intensity was normalized using a MgO:Mn^2+^ standard, and the radical concentration was estimated from the integrated area of the spectra, which is directly proportional to the double integral of the absorption curve.^58^ EPR spectra of each sample are presented in Figure S13 of the Supporting Information, together with the deconvolution of the spectra of sample NF4, where the distinct ROS produced are described. Here, only ^•^OOH and ^•^OH radicals are discussed, as other radicals are produced from secondary reactions and do not directly affect the efficiency of the samples in AOPs. A control experiment in the absence of NFs is also presented in the Supporting Information in Figure S14.

**Figure 6:**
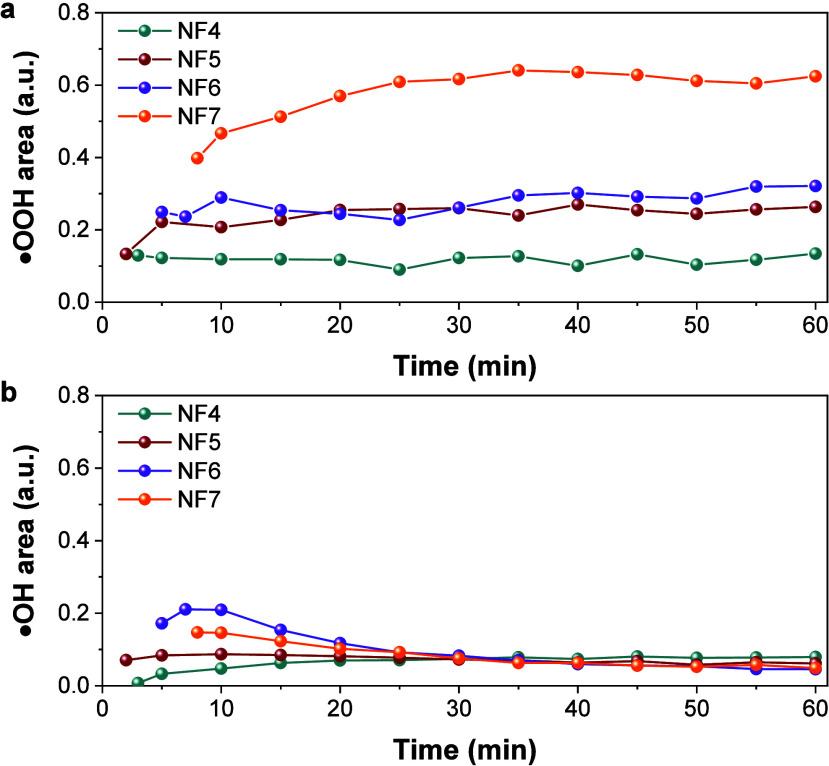
Time evolution of reactive oxygen species generated by nanoflowers monitored by EPR spectroscopy in the presence of H_2_O_2_: (a) hydroperoxyl radicals (^•^OOH) and (b) hydroxyl radicals (^•^OH). The EPR signal intensity is represented as the integrated area normalized to the Mn^2+^ signal (MgO:Mn^2+^ reference). Undoped samples correspond to NF4 (nonsintered) and NF5 (sintered), while cobalt-doped counterparts correspond to NF6 (nonsintered) and NF7 (sintered).

The first set of curves (Figure 6a) corresponds to the evolution of ^•^OOH radicals. The Co-free, nonsintered sample (NF4) displayed the lowest radical yield, whereas cobalt incorporation (NF6) markedly increased ^•^OOH generation. This enhancement can be attributed to Co^2+^ ions creating additional electronic states that facilitate charge transfer and reduce recombination, favoring superoxide formation that evolves into ^•^OOH.^59,60^ In parallel, cobalt doping and thermal treatment modify surface defect chemistry and oxygen vacancy distribution, introducing additional catalytic sites that promote radical generation. These modifications enhance surface reactivity, enabling more efficient adsorption and transformation of H_2_O_2_ and reactive intermediates.^61^ Moreover, cobalt ions redox cycling (Co^2+^/Co^3+^) exhibits a higher redox potential (1.30 V) than Fe^2+^/Fe^3+^ (0.771 V), thereby providing stronger driving forces for radical production. In general, catalytic activity depends on the nature of divalent metal M, redox potential, synthesis method, annealing temperature, etc.^62,63^

The second set of curves (Figure 6b) shows the evolution of ^•^OH radicals. In all cases, their contribution was lower compared to ^•^OOH, but similar trends were observed. NF4 generated the lowest amount, NF6 exhibited increased radical production due to cobalt doping, and the sintered samples (NF5 and NF7) displayed further enhancement. Notably, NF7 again showed the highest radical yield, confirming the synergistic effect of structural ordering and dopant incorporation.

Sintering also exerts a clear influence: NF5 (undoped, sintered) exhibited higher ^•^OOH production compared to NF4, while NF7 (Co-doped, sintered) reached the maximum yield, confirming the combined effect of crystallinity improvement and cobalt incorporation. This behavior can be rationalized by the structural and electronic modifications induced during the sintering step. On the one hand, sintering promotes partial fusion of the primary cores, leading to enhanced crystallinity, reduction of lattice disorder, and reorganization of oxygen vacancies, which together create more stable and catalytically active sites with improved electron mobility. Recent work on α-Fe_2_O_3_ has demonstrated that sintering is driven by the diffusion of surface atoms, especially Fe, producing surface enrichment and catalytically more active sites, while simultaneously increasing crystallinity and transforming small pores into larger and more stable channels.^64^ These well-ordered domains provide a more efficient framework for charge separation and reactive intermediate formation in the undoped sample (increasing surface charge with thermal annealing in NF5), thus boosting ^•^OOH yields (see surface charge profiles in Figure S6 of the Supporting Information). Interestingly, cobalt-doped samples do not display further increase in surface charge density after annealing, suggesting that Co incorporation already provides an optimal number of active sites and that the primary benefit of the thermal treatment in NF7 is the enhancement of crystallinity and cooperative electron transport. The combination of these effects results in NF7 delivering the highest radical production among all tested samples.

Table S2 summarizes the maximum areas resulting from the different free radicals generated by NF4–7 samples. Overall, both the thermal treatment during synthesis and the incorporation of cobalt markedly enhance the efficiency of ROS generation in magnetite nanoflowers. These effects can be attributed to improved crystallinity and the creation of more stable and catalytically active sites.^65^ Importantly, beyond these intrinsic properties, the kinetics of ROS-mediated AOPs can be further enhanced under magnetic hyperthermia conditions by applying an AMF. This dual functionality highlights the relevance of the present nanoflowers, as they combine efficient ROS generation with high heating capability. Such features make them particularly promising for magnetically assisted catalytic processes, where temperature plays a key role in accelerating reaction rates, in addition to their potential use in magnetic hyperthermia.^19,66–69^

### 2.5 X-ray Absorption Spectroscopy Analysis

To further confirm that the catalytic activity is due to a specific electronic configuration, we performed X-ray absorption spectroscopy (XAS) at the Fe K-edge on the NF samples at the BM23 beamline of the ESRF synchrotron (Grenoble, France). Before the acidic treatment (BAT), for the nondoped samples grown under different heating stages (Figure 7), electronic analysis confirmed that NF synthesized during the first heating stage (NF4-BAT) exhibited an absorption edge corresponding to mixed Fe^2+^/Fe^3+^ states, associated with a proportion of magnetite/maghemite phases when compared with the references (Figure 7a). In contrast, the second heating step (NF5-BAT) revealed a predominantly Fe^3+^ profile, consistent with the maghemite phase, which was maintained after acidic treatment (Figure 7c). Moreover, NF5-BAT exhibited an increased white line intensity relative to NF4-BAT, reflecting the higher Fe^3+^ contribution and a more ordered coordination environment, consistent with improve crystallinity (Figure 7a, top).

**Figure 7:**
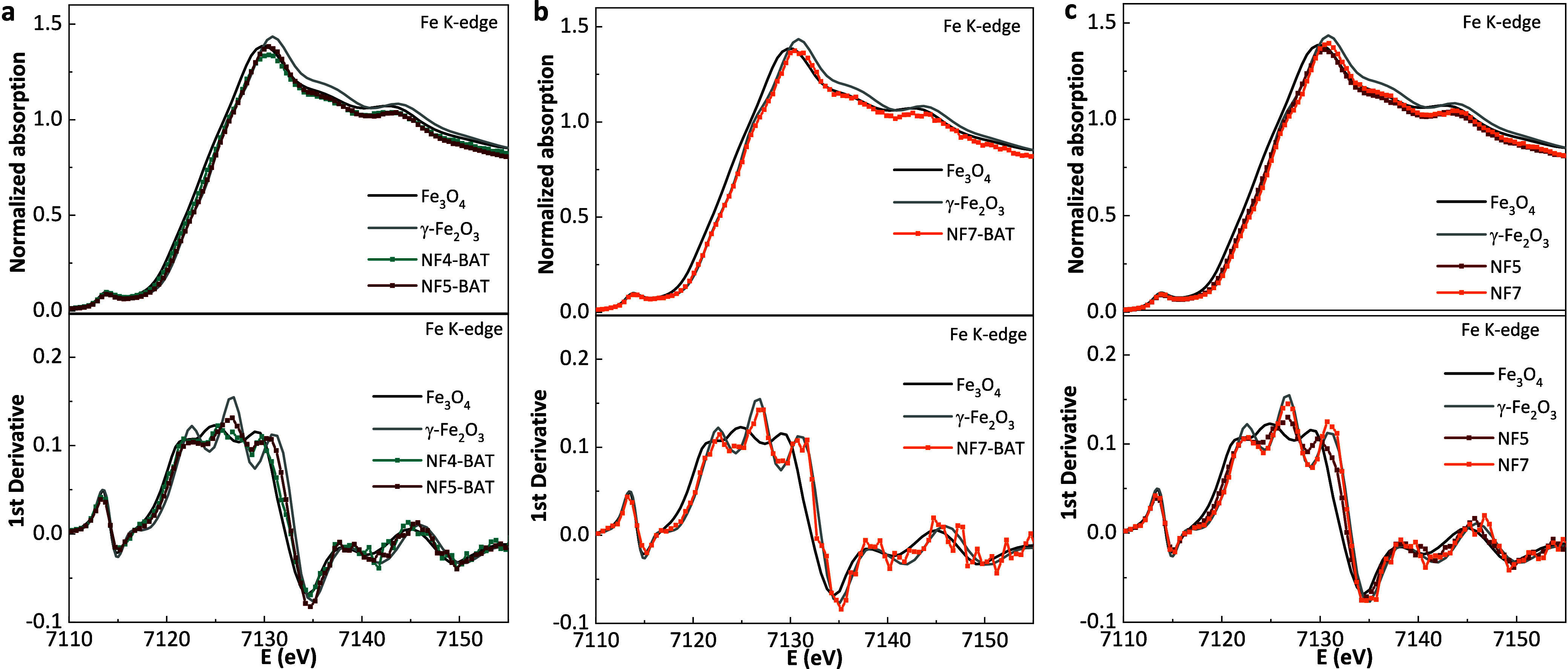
XANES spectra at the Fe K-edge (top) and corresponding first derivatives (bottom) for: (a) the effect of thermal treatment, comparing NF4 and NF5 prior to acidic treatment (BAT); (b) the effect of cobalt incorporation, shown for NF7 (BAT); and (c) the effect of acidic treatment: NF5 and NF7. In all cases, spectra are compared with Fe_3_O_4_ and γ-Fe_2_O_3_ references. NF4 and NF5 correspond to undoped samples (nonsintered and sintered, respectively), while NF7 corresponds to sintered Co-doped samples sintered.

Finally, the Co-doping effect was also investigated using the XAS technique (Figure 7b). For this purpose, NF7-BAT (Co-doped) and NF5-BAT (undoped) were compared. NF7-BAT also exhibited a dominant Fe^3+^ profile after Co^2+^ doping, with the absorption edge shifting toward energies characteristic of Fe^3+^ due to the decreased fraction of any available Fe^2+^ from the initial heating rate (NF4) upon substitution by Co^2+^.

Regarding catalytic activity: XANES at the Fe K-edge shows an increase in the energy edge and white-line intensity from NF4-BAT to NF5/NF7-BAT, indicating a higher Fe^3+^ fraction and improved local order compared with NF4-BAT. These parameters correlate positively with the ^•^OOH yield, supporting that crystallographic coherence and an oxidized Fe framework, complemented by Co redox centers, facilitate H_2_O_2_ activation, as shown in Figure 6.

## 3 Conclusion

This work demonstrates a transformative approach for the design and synthesis of iron oxide nanoflowers, bridging the gap between fundamental nanomaterials research and scalable application-oriented production. We successfully translated the conventional polyol-based autoclave synthesis into a microwave-assisted route, preserving the crystallographic order of the multicore assemblies while dramatically reducing the reaction time and exhibiting excellent reproducibility. This advance not only renders the process more energy efficient and sustainable but also establishes a realistic pathway toward industrial scalability.

Equally important, we identified and defined critical parameters controlling nucleation, growth, and crystallographic alignment, offering valuable mechanistic insights into how ordered multicore architectures can be rationally engineered. Such knowledge expands the toolbox of synthetic chemistry for complex oxide nanostructures, providing guidelines that extend beyond those of the specific system studied here.

Our work also demonstrates how magnetic anisotropy can be deliberately engineered during synthesis by combining controlled thermal treatment with cobalt incorporation. By linking these structural choices to the resulting electronic and magnetic properties, we show that it is possible to simultaneously boost the catalytic activity, radical generation, and magnetic hyperthermia performance. The nanoflowers obtained were designed to achieve high heating efficiency under alternating magnetic fields, underscoring their value as multifunctional platforms with strong potential in applications where both ROS generation and magnetic heating are essential, such as AOPs or biomedical treatments.

## 4 Experimental Section

### 4.1 Chemical Reagents and Analysis

Iron­(III) chloride hexahydrate (FeCl_3_·6H_2_O, ≥98%), iron­(II) chloride (FeCl_2_, ≥98%), sodium hydroxide (NaOH, ≥98%), *N*-methyldiethanolamine (NMDEA, 99%), diethylene glycol (DEG, 99%), cobalt­(II) sulfate heptahydrate (CoSO_4_·7H_2_O ≥99%), ethyl acetate (≥99.8%), iron nitrate (Fe­(NO_3_)_3_·9H_2_O) and nitric acid (65%, HNO_3_) were purchased from Sigma-Aldrich. Ethanol (≥99%) was purchased from Scharlau.

The iron concentration of the NF samples was assessed using inductively coupled plasma optical emission spectrometry (ICP-OES) conducted with Optima 2100 DV PerkinElmer equipment. For this purpose, 25 μL of the nanoparticle suspension was digested in 1 mL of aqua regia at 90 °C overnight. Subsequently, the solution was diluted to a final volume of 25 mL with distilled water.

### 4.2 Synthesis of Iron Oxide Nanoflowers

The NFs samples were prepared based on a previously established solvothermal method in an autoclave, with necessary modifications tailored to adapt the synthesis process for microwave heating.^19,31^ Briefly, a solvent mixture of DEG and NMDEA (50:50 w/w) was prepared, and 0.1 g of NaOH was added to 6.4 g of the solvent and mixed by ultrasonication for 7 min at 70 °C. Simultaneously, 6.4 × 10^–4^ mol of FeCl_3_·6H_2_O (0.173 g) and 3.1 × 10^–4^ mol of FeCl_2_ (0.039 g) were added to 12.8 g of the solvent mixture and magnetically stirred for 45 min. Finally, the NaOH mixture was added to the mixture of iron salts in a G30 microwave vial and immediately placed inside the microwave (Monowave 300), where the reaction took place, considering specific conditions for heating ramp, temperature, and reaction time.

After each synthesis, the precipitates were magnetically recovered and washed with a mixture of ethanol and ethyl acetate (50:50 v/v) five times. To increase colloidal stability, NF samples were subjected to an acidic and oxidation treatment following a previously described protocol.^19^ For this, the precipitates obtained were washed again with 10 mL of nitric acid (10%), and the supernatant was magnetically separated. Then, NF samples were redispersed in 10 mL of distilled water and magnetically stirred with Fe­(NO_3_)_3_·9H_2_O (1 M in the final solution) for 30 min at 90 °C. The supernatant was magnetically separated and allowed to cool to room temperature. After cooling, NF samples were washed again with 10 mL of nitric acid (10%), and the supernatant was magnetically separated. The obtained precipitates were washed 5 times with ethanol, and the mixture was dispersed in distilled water.

To analyze the effect of the reaction time, three different experiments were performed considering a fast heating ramp of 4 °C s^–1^, a temperature of 220 °C, and reaction times of 3, 5, and 10 min, labeling the last sample as NF1. Consequently, the reaction time was fixed at 10 min, and the effect of the heating ramp was analyzed: 0.2 °C s^–1^ (NF2) and 0.03 °C s^–1^ (NF3). To study the sintering of the NF samples, the heating ramp was fixed at 0.2 °C s^–1^, while the reaction time was extended to 20 min, and the obtained sample was labeled as NF4.

### 4.3 Thermal Ordered Assembly

To ensure that the NFs samples reached the specific stage in the formation mechanism where crystallographic alignment occurs, the same microwave conditions used for NF4 were applied, with the addition of an extra heating step to induce slight fusion of the cores. This step facilitated the alignment of the cores within the nanostructure, enhancing their structural coherence and collective magnetic response. After preparing the precursor mixture in a G30 vial, the synthesis was conducted in the microwave reactor, considering a two-step heating protocol. The first step involved a heating rate of 0.2 °C s^–1^ to 220 °C, which was maintained for 20 min to support the nucleation, aggregation, and growth of the cores. In the second step, the temperature was increased at a slower rate of 0.1 °C s^–1^ to 250 °C and held for 1 h, allowing slight core sintering to promote alignment. After synthesis, the sample was labeled NF5 and underwent washing and treatment following the previously established protocol to remove residual precursors and byproducts.

### 4.4 Cobalt Incorporation

To investigate the effect of cobalt doping on the magnetic properties of ferrite NF, cobalt was introduced by adjusting the precursor amounts while following the same synthesis conditions as NF4 in the microwave reactor. Specifically, CoSO_4_·7H_2_O was introduced in molar proportions of 0.1 relative to the total metal content in the reaction mixture, replacing 20 wt % of the amount of FeCl_2_ by the Co salt. The reaction was conducted in a microwave reactor with a single heating stage: a heating rate of 0.2 °C s^–1^ to 220 °C, held for 20 min. After synthesis, the sample was labeled NF6. Additionally, a second heating step was introduced, under the same conditions applied for NF5, and the sample was labeled as NF7. After syntheses, the samples were washed and treated with acid following the same protocol as previously described to ensure sample stability, purity, and consistency.

The synthetic parameters for each nanoflower sample are summarized in Table 1, including the specific heating profiles, holding times, and annealing steps applied during microwave-assisted synthesis.

### 4.5 Reproducibility

To assess the reproducibility of the synthetic protocol, a total of 7 independent batches of NFs were synthesized. For this purpose, a concentrated stock solution was prepared by scaling up the amounts of solvents and precursors to yield 126 mL of reaction mixture. This solution was then evenly distributed into seven individual vials. The synthesis was performed in automated mode using the autosampler function of the microwave reactor, which employs a robotic arm to sequentially carry out each synthesis under identical conditions. Upon completion, each NF batch was subjected to five washing cycles using an acetate–ethanol mixture to remove residual precursors and byproducts and subjected to the acidic treatment previously mentioned, followed by final dispersion in water to obtain stable colloidal suspensions.

### 4.6 Structural and Morphological Characterization

The structural characterization of the synthesized nanoparticles was carried out using X-ray diffraction (XRD), employing a Bruker D8 Advance diffractometer. Data were collected in the 2θ range of 10–70°, enabling the identification of crystalline phases and estimation of average crystallite sizes. The diffraction patterns were analyzed using EVA software, and the crystallite size was calculated by fitting the most intense peaks to the Scherrer equation (*K* = 9): *D* = *K*λ/(*B*·cos θ), where *D* is the crystallite size, λ is the X-ray wavelength, *B* is the full width at half-maximum (FWHM), and θ is the Bragg angle. Instrumental broadening was corrected using a standard reference sample.

Morphological and dimensional analyses of the nanoparticles were performed by transmission electron microscopy (TEM), using a JEOL-JEM 1010 microscope operating at an acceleration voltage of 100 keV and equipped with a Gatan Orius 200 SC digital camera. High-resolution TEM (HRTEM) was performed using a CM200 microscope at 200 kV. Representative micrographs were processed using ImageJ software. A minimum of 200 individual nanoparticles were manually measured from multiple micrographs, and the resulting data were fitted to a log-normal function to extract the mean particle size and its dispersion.

### 4.7 Magnetic Characterization

Magnetic characterization was conducted with a SQUID magnetometer (Quantum Design MPMS-3), which allows precise measurements across a broad range of magnetic field strengths and temperatures, operating in DC. Hysteresis loops were recorded at both room temperature (290 K) and cryogenic conditions (5 K), using applied magnetic fields in the range of ±4000 kA m^–1^. These measurements provided insights into the saturation magnetization, coercivity, and temperature-dependent magnetic behavior of the nanoparticle ensembles. A known quantity of the powder sample was compressed into pellets for measurement, and the data are given in kg of nanoparticles.

Ferromagnetic resonance (FMR) experiments were carried out on a Bruker ESP300 spectrometer operating in the Q-band (39.9 GHz). Measurements were performed at room temperature with a 100 kHz modulating frequency and 10 G amplitude. For sample preparation, the nanoflowers were dispersed in an ethyl cyanoacrylate adhesive (instant glue) to immobilize their orientation and minimize interparticle interactions.

### 4.8 Synchrotron X-Ray Absorption Spectroscopy (XAS)

X-ray absorption spectra were collected in fluorescence mode at the BM23 beamline of the ESRF synchrotron (Grenoble, France). Measurements at the Fe K-edge (7112 eV) were performed at room temperature. Energy calibration was carried out using the Fe foil K-edge, with γ-Fe_2_O_3_ and Fe_3_O_4_ serving as reference compounds. Spectra were normalized to a unit edge jump through linear pre-edge and polynomial postedge fitting for background subtraction.^100^ Data analysis was performed using the Athena software package.^70^

### 4.9 Magnetic Hyperthermia

To evaluate the heating performance of the nanoflowers under an alternating magnetic field (AMF), the specific absorption rate (SAR) was determined, which is a fundamental parameter that quantifies the power dissipated per unit mass of magnetic material. SAR values were obtained using an FIVES CELES AMF induction system (model no. 12118 M01, France) equipped with a water-cooled copper coil (50 mm in diameter, six turns).

Samples were placed in Eppendorf tubes positioned at the center of the coil to ensure uniform field exposure. Experiments were conducted at frequencies ranging from 100 to 200 kHz and magnetic field amplitudes between 8 and 48 kA m^–1^. Thermal insulation of the coil and thermostat control allowed for a stable baseline temperature in the absence of magnetic excitation.

Nanoflowers were dispersed at a concentration of 1 mg mL^–1^ in aqueous solution and subjected to brief ultrasonication before each measurement to ensure homogeneous suspension. The initial temperature was adjusted to 21 °C using a thermostatic control unit, and temperature evolution was monitored in real time using a fiber optic sensor inserted directly into the sample. Each measurement lasted 5 min: the magnetic field was turned on at 60 s, maintained for 300 s, and turned off at 360 s. The SAR was calculated from the initial linear slope of the temperature vs time curve, according to the equation: SAR = (*C*_*p*_/*c*)­(d*T*/d*t*), where *C_p_* is the specific heat capacity of the system (in J L^–1^ K^–1^), *c* is the concentration of the material in the suspension (in g L^–1^), and d*T*/d*t* is the initial slope of the temperature increase (in °C s^–1^). For aqueous dispersions at low nanoparticle concentration, *C_p_* was approximated by the specific heat capacity of water (4185 J L^–1^ K^–1^).

### 4.10 Free Radical Production for AOPs

The ability of the synthesized nanoflowers to generate ROS was evaluated in the context of AOPs by electron paramagnetic resonance (EPR) spectroscopy. Measurements were carried out in the X-band (9.5 GHz) at room temperature using a BRUKER ELEXSYS II-E500 spectrometer. To detect and quantify transient free radicals, 5,5-dimethyl-1-pyrroline *N*-oxide (DMPO) was used as a spin-trapping agent and dissolved in dimethyl sulfoxide (DMSO) with a concentration of 0.17 g mL^–1^. A modulation frequency of 100 kHz and a field modulation amplitude of 3 G were applied. As a reference for signal normalization and semiquantitative comparison, the stable resonance of Mn^2+^ defects in a MgO crystal (MgO:Mn^2+^) was used.

For each experiment, the NFs were suspended at a concentration of 1 mg mL^–1^ in 200 μL of acetate buffer (pH 4.5) within a quartz EPR tube. Subsequently, 50 μL of the DMPO/DMSO solution and 10 μL of a 30% hydrogen peroxide solution were sequentially added to initiate the radical-generating reaction. EPR spectra were acquired at 5 min intervals to monitor the evolution of radical species over time.

Spectral deconvolution and quantification were performed using Bruker SpinFit software, which enables multicomponent fitting based on hyperfine splitting parameters. Radical signatures were identified and assigned using the NIEHS spin trap database (https://tools.niehs.nih.gov/stdb/index.cfm/spintrap/), allowing discrimination between hydroxyl radicals, superoxide, and other species. This methodology provided a robust framework for assessing the catalytic potential of the nanoflowers in oxidative environments relevant to environmental remediation technologies.

## Supplementary Material



## Data Availability

The data that support the findings of this study are available from the corresponding author upon reasonable request.

## Notes

Synthesis of iron oxide nanoflowers via a microwave-assisted method at 220 °C for 10 min using a heating ramp of 0.05 °C s^–1^: (a) TEM image, (b) particle and core size distribution, and (c) XRD pattern (Figure S1); synthesis of iron oxide nanoflowers via a microwave-assisted method at 220 °C for 120 min using a heating ramp of 0.2 °C s^–1^: (a) TEM image, (b) particle and core size distribution, and (c) XRD pattern (Figure S2); microwave temperature profile during the synthesis (Figure S3); representative HRTEM images and corresponding selected area electron diffraction (SAED) patterns of iron oxide nanoflowers at different stages of structural evolution: (a, b) NF4 sample showing a multicore structure with limited crystallographic coherence and a ring-like SAED pattern characteristic of randomly oriented nanocrystals, (c, d) NF5 sample after thermal treatment, displaying a more compact morphology and enhanced crystallographic alignment, as evidenced by the appearance of discrete diffraction spots superimposed on diffuse rings in the SAED pattern, indicative of mesocrystalline ordering (Figure S4); different degrees of sintering of nanoflowers during the second heating stage at 250 °C: (a) TEM image of NFs after 5 min, (b) size distribution, (c) most intense peak of the XRD pattern, (d) TEM image after 30 min, (e) size distribution, and (f) most intense peak of the XRD pattern (Figure S5); hydrodynamic size distribution (in intensity) obtained by DLS for NF5 sample (Figure S6); surface charge profiles (as a function of pH) for (a) NF4 (undoped, nonsintered), (b) NF5 (undoped, sintered), (c) NF6 (Co-doped, nonsintered), and (d) NF7 (Co-doped, sintered) (Figure S7); TEM images and particle size distribution of NF4 nanoflowers synthesized automatically using a robotic arm and carousel in the Anton Paar Monowave microwave system to evaluate reproducibility (Figure S8); X-ray diffraction patterns of NF4 nanoflowers synthesized automatically using a robotic arm and carousel in the Anton Paar Monowave microwave system (Figure S9); DLS curves of different NF4 batches: hydrodynamic size distributions in (a) intensity and (b) number (Figure S10); magnetic hysteresis loops at 290 K of NF4 batches synthesized automatically using the robotic microwave system (Figure S11); heating curves of NF4 batches as a function of time under an alternating magnetic field of 24 kA m^–1^ and a frequency of 200 kHz (Figure S12); EPR spectra of NF4 and deconvolution of ^•^OOH and ^•^OH radical signals (Figure S13); structural, magnetic, colloidal, and heating parameters of NF4 batches synthesized by the microwave-assisted method, including particle size, core size, crystal size, coercivity, susceptibility, magnetization at 290 K, specific absorption rate (SAR), and yield (Table S1); control experiment for ROS detection monitored by EPR spectroscopy in the absence of nanoflowers (blank) (Figure S14) (PDF)
