# Supplementary material for: Designed to Heat and React: Fast Microwave-Engineered Iron Oxide Nanoflowers with Controlled Anisotropy for Magnetic Induction-Assisted Oxidation Processes
Source: ACS Appl Nano Mater. 2026 May 8;9(20):9497–512. doi: 10.1021/acsanm.6c01066 (PMC13206268; doi:10.1021/acsanm.6c01066)
Supplement: Supplementary file 1 [file an6c01066_si_001.pdf]

Supporting Information

Designed to Heat and React: Fast Microwave-Engineered Iron Oxide Nanoflowers with Controlled Anisotropy for Magnetic Induction-assisted Oxidation Processes

Rafael Herrera-Aquino[a], *Nahuel Nuñez,*[b,c,d], *Raúl Magro*[a], *Sabino Veintemillas-Verdaguer*[a], *Elin L. Winkler*[b,c,d], *Ana Espinosa*[a], *María del Puerto Morales*[a,*], *Alvaro Gallo-Cordova*[a,*]

[a] Instituto de Ciencia de Materiales de Madrid, ICMM/CSIC, C/Sor Juana Inés de la Cruz 3, 28049 Madrid, Spain

[b] Departamento Magnetismo y Materiales Magnéticos, Gerencia de Física, Centro Atómico Bariloche, Av. Bustillo 9500, (8400) S. C. de Bariloche (RN), Argentina

[c] Instituto de Nanociencia y Nanotecnología (CNEA-CONICET), Nodo Bariloche, Av. Bustillo 9500, (8400) S. C. de Bariloche (RN), Argentina.

[d] Instituto Balseiro, CNEA-UNCuyo, Av. Bustillo 9500, 8400 San Carlos de Bariloche, Río Negro, Argentina

*Corresponding authors' e-mail: puerto@icmm.csic.es (M.P.M) and alvaro.gallo@csic.es (A.G.-C.)

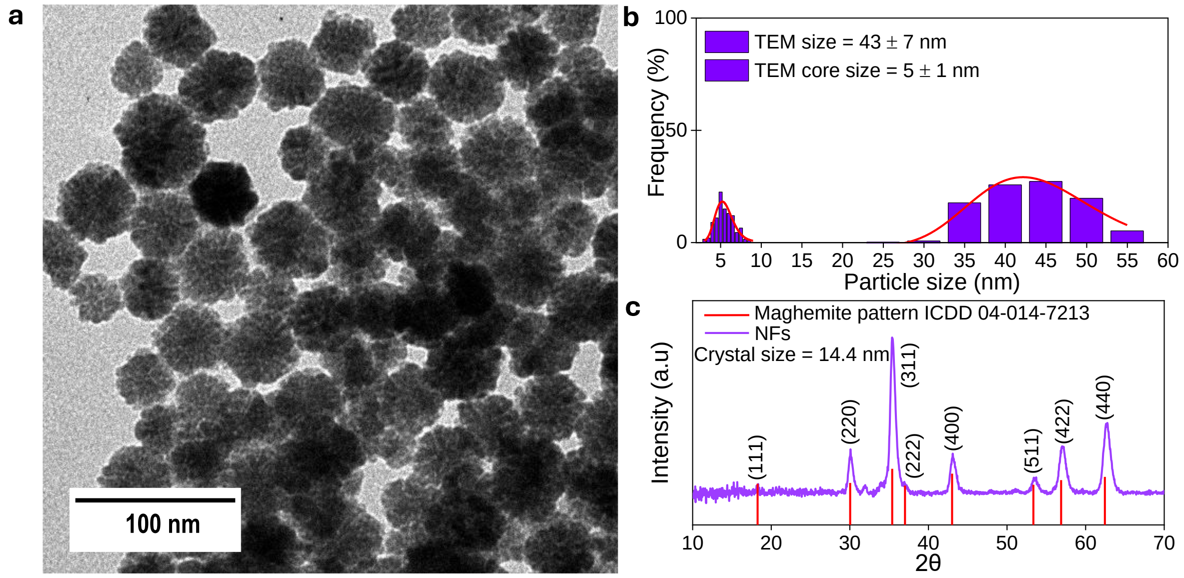

Figure S1. Synthesis of iron oxide nanoflowers via a microwave-assisted method at 220 °C for 10 minutes, using a heating ramp of 0.05 °C/s: a) TEM image, b) Particle and core size distribution and c) XRD pattern.

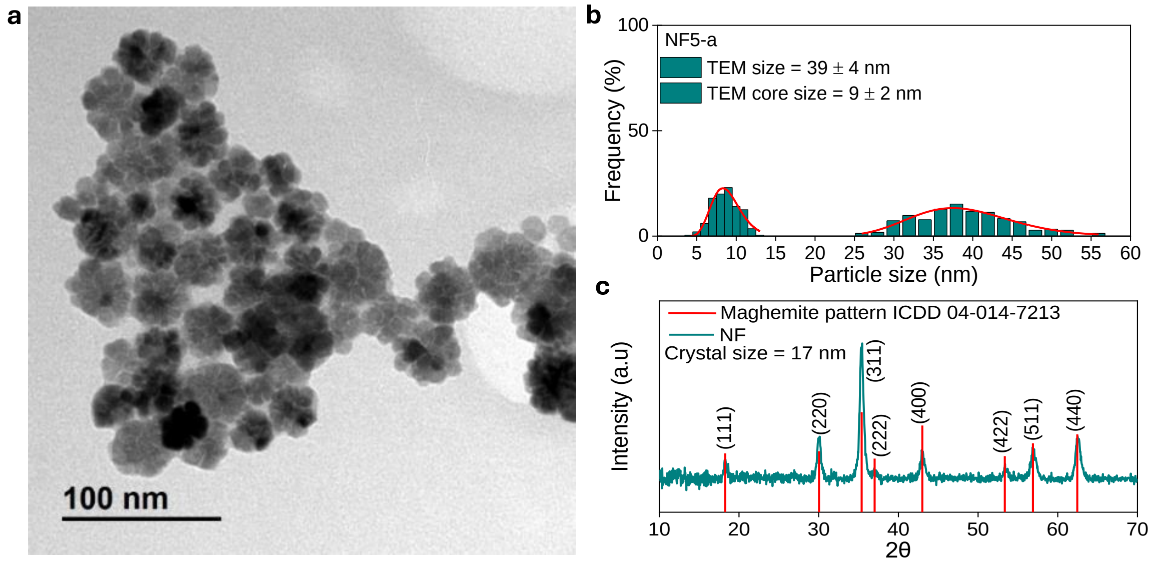

Figure S2. Synthesis of iron oxide nanoflowers via a microwave-assisted method at 220 °C for 120 minutes, using a heating ramp of 0.2°C/s: a) TEM image, b) Particle and core size distribution and c) XRD pattern.

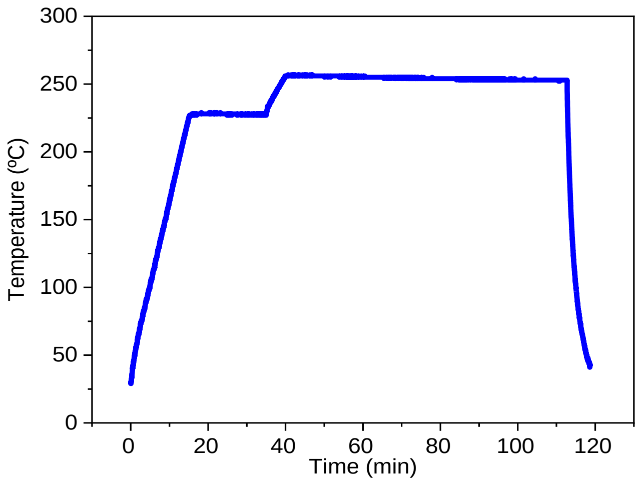

Figure S3. Microwave profile temperature

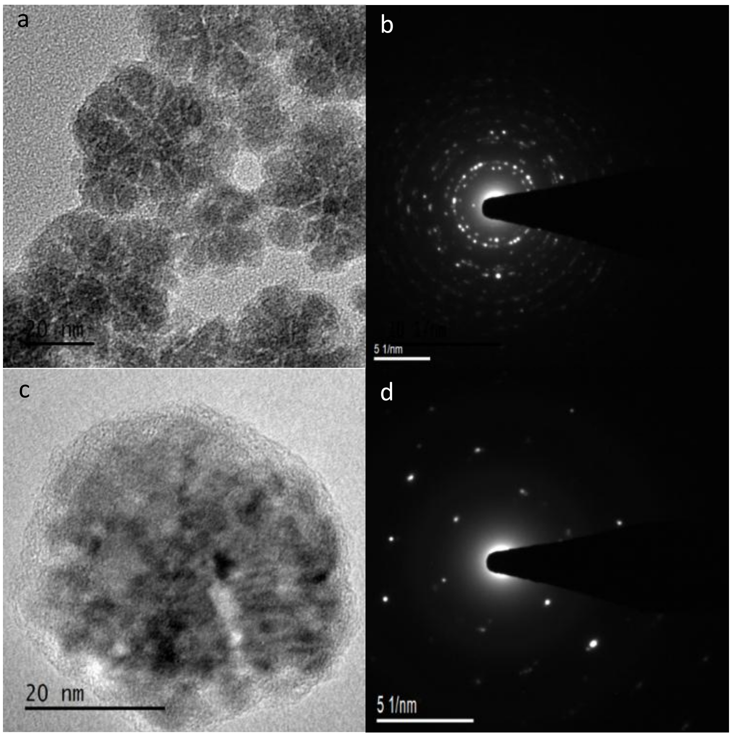

Figure S4. Representative HRTEM images and corresponding selected area electron diffraction (SAED) patterns of iron oxide nanoflowers at different stages of structural evolution. (a,b) NF4 sample showing a multicore structure with limited crystallographic coherence and a ring-like SAED pattern characteristic of randomly oriented nanocrystals. (c,d) NF5 sample after thermal treatment, displaying a more compact morphology and enhanced crystallographic alignment, as evidenced by the appearance of discrete diffraction spots superimposed on diffuse rings in the SAED pattern, indicative of mesocrystalline ordering.

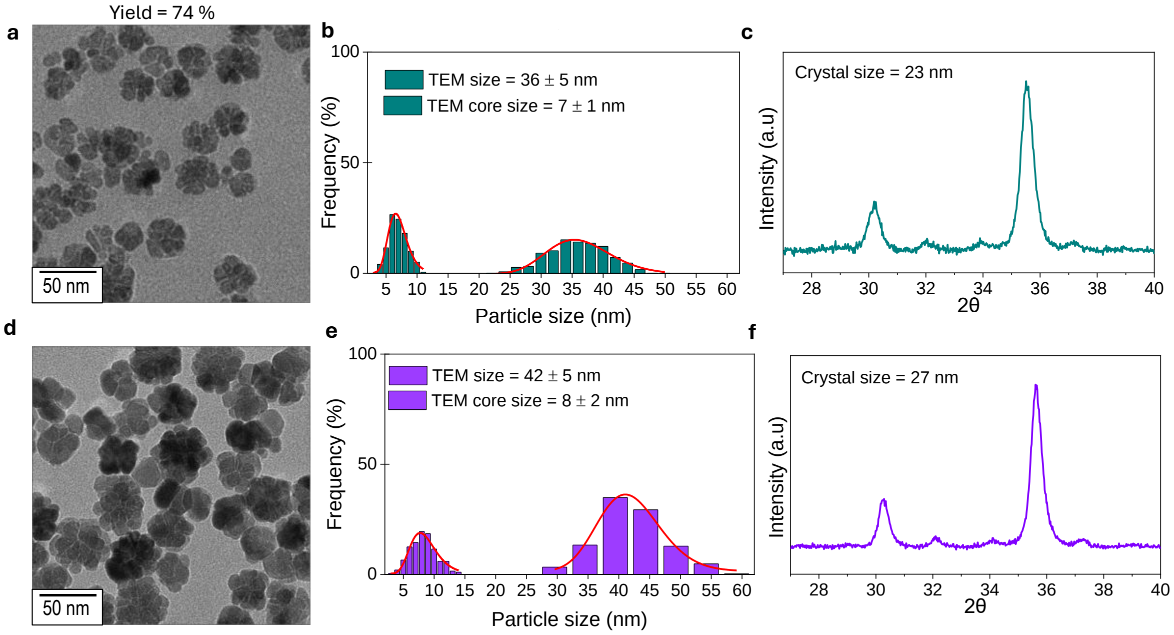

Figure S5. Different degrees of sintering of nanoflowers: a) TEM image of NFs subjected to 5 minutes of the second heating stage (250 °C) with their corresponding size distribution (b) and the most intense peak from X-ray pattern (c). d) TEM image of NFs subjected to 30 minutes of the second heating step (250 °C) with their corresponding size distribution (e) and the most intense peak from X-ray pattern (f).

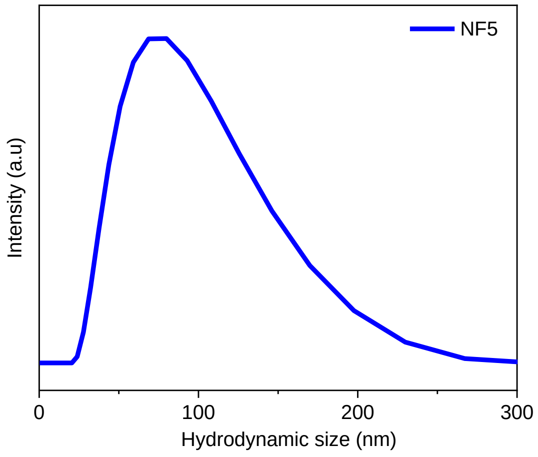

Figure S6. Hydrodynamic size distribution (in intensity) obtained by DLS for NF5 sample.

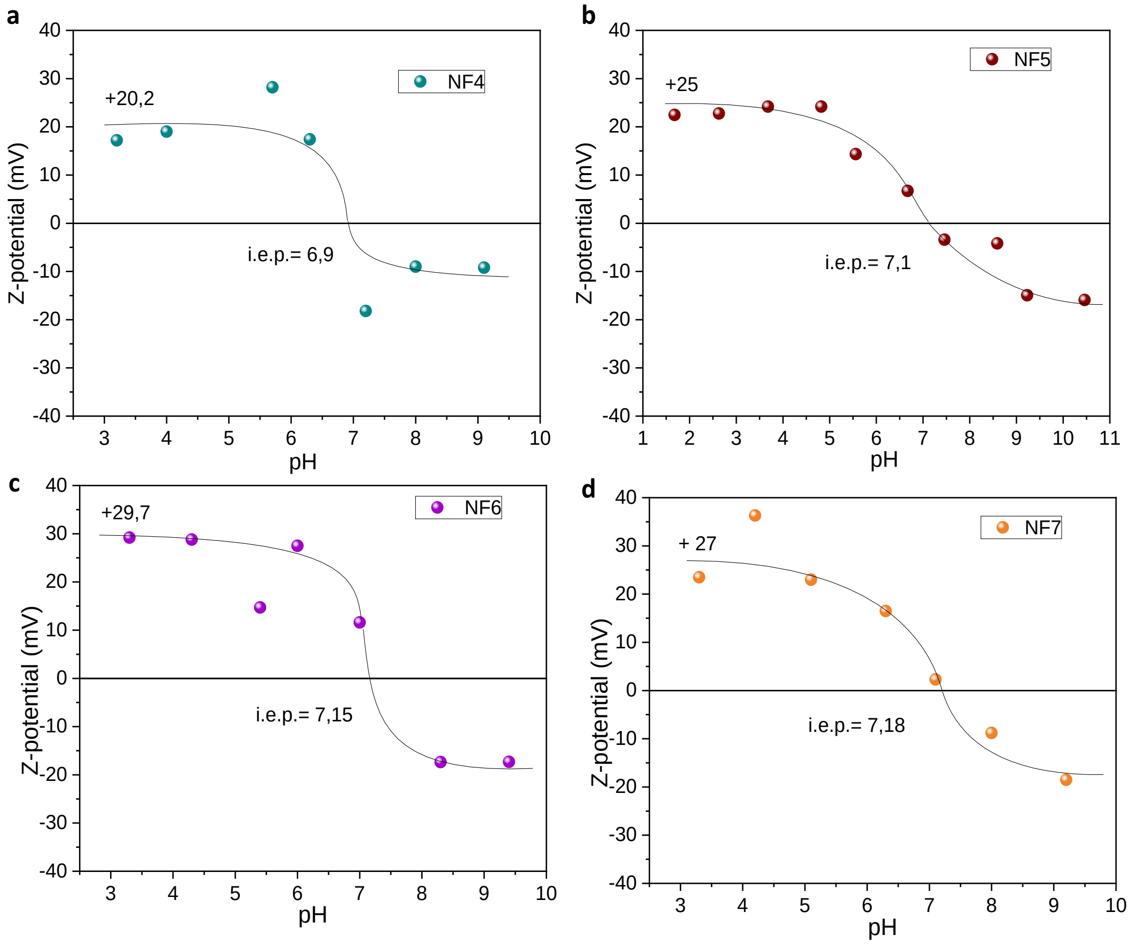

Figure S7. Surface charge profiles (as a function of pH) for (a) NF4 (undoped, non-sintered), (b) NF5 (undoped, sintered), (c) NF6 (Co-doped, non-sintered), and (d) NF7 (Co-doped, sintered). Sintering (NF5) increases the surface charge density over a wide pH range, indicating a higher density of surface hydroxyl groups and redox-active Fe^{2+}/Fe^{3+} sites, which correlates with enhanced catalytic activity. In contrast, Co-doped samples (NF6, NF7) exhibit similar charge densities regardless of the thermal treatment, suggesting that cobalt incorporation already maximizes the density of accessible surface sites. Continued lines are included as guide to the eye.

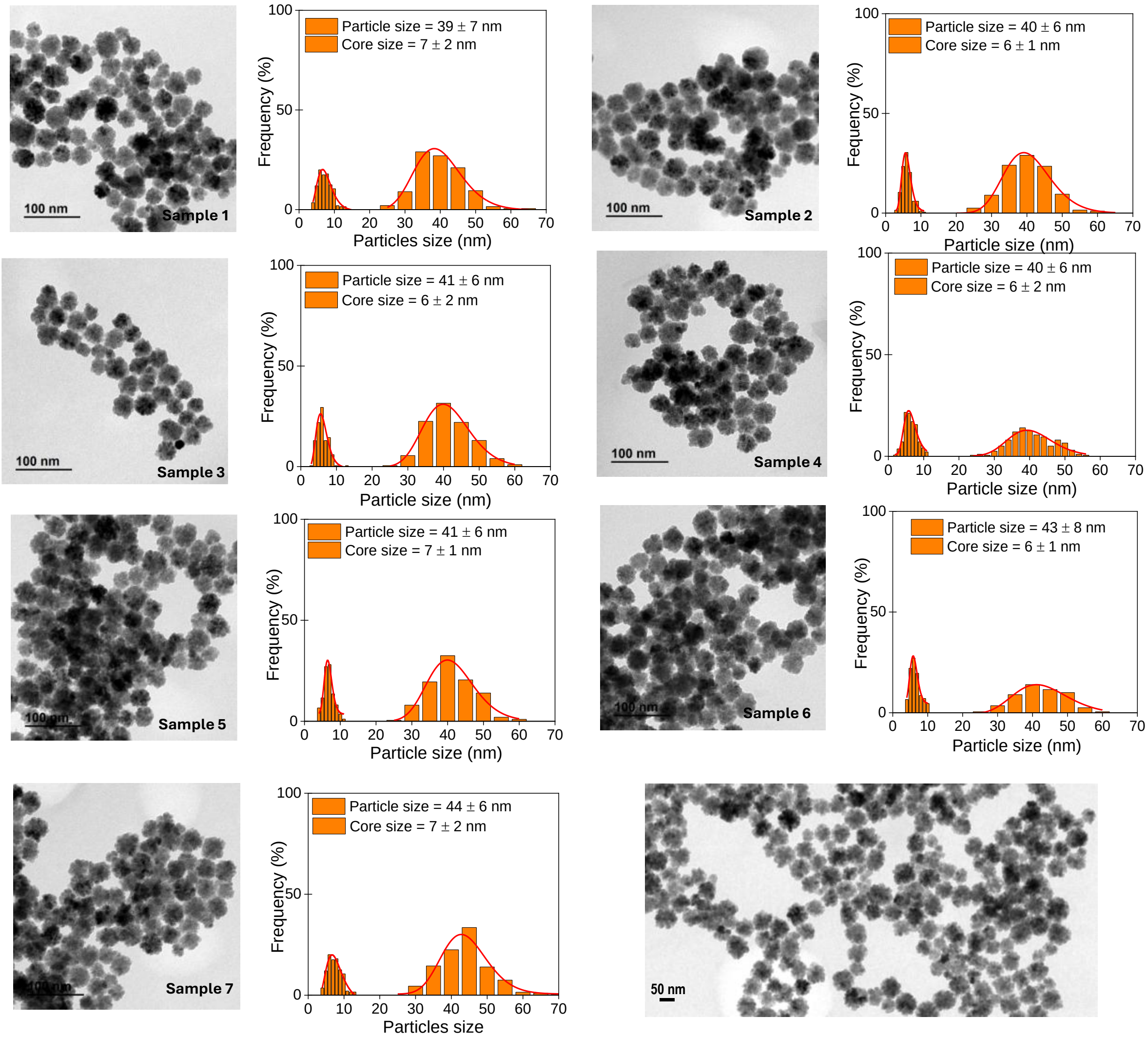

Figure S8. TEM images and particle size distribution of NF4 nanoflowers synthesized automatically by an arm robot and carrousel within the Anton Paar® Monowave Microwave to check their reproducibility.

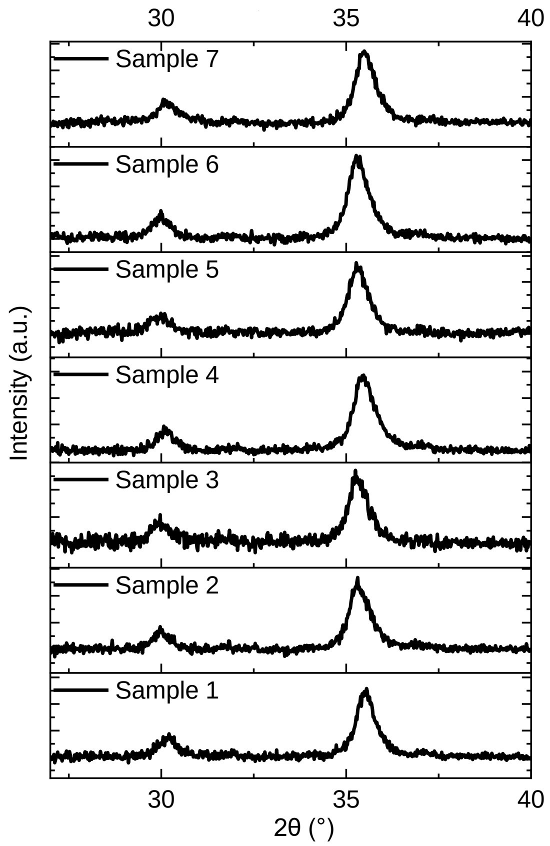

Figure S9. X-ray diffraction patterns of NF4 nanoflowers synthesized automatically by an arm robot and carrousel within the Anton Paar® Monowave Microwave to check their reproducibility.

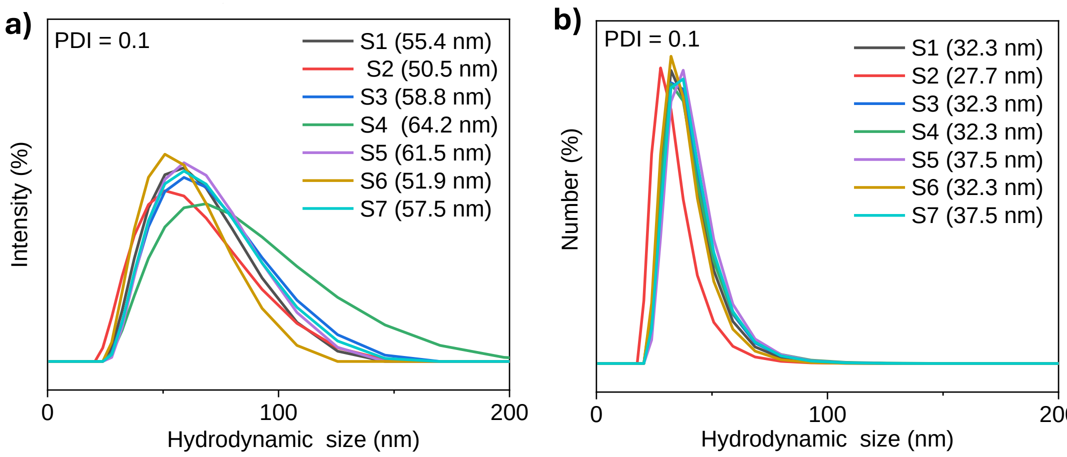

Figure S10. DLS curves: Hydrodynamic size distributions in intensity (a) and number (b) of different NF4 batches.

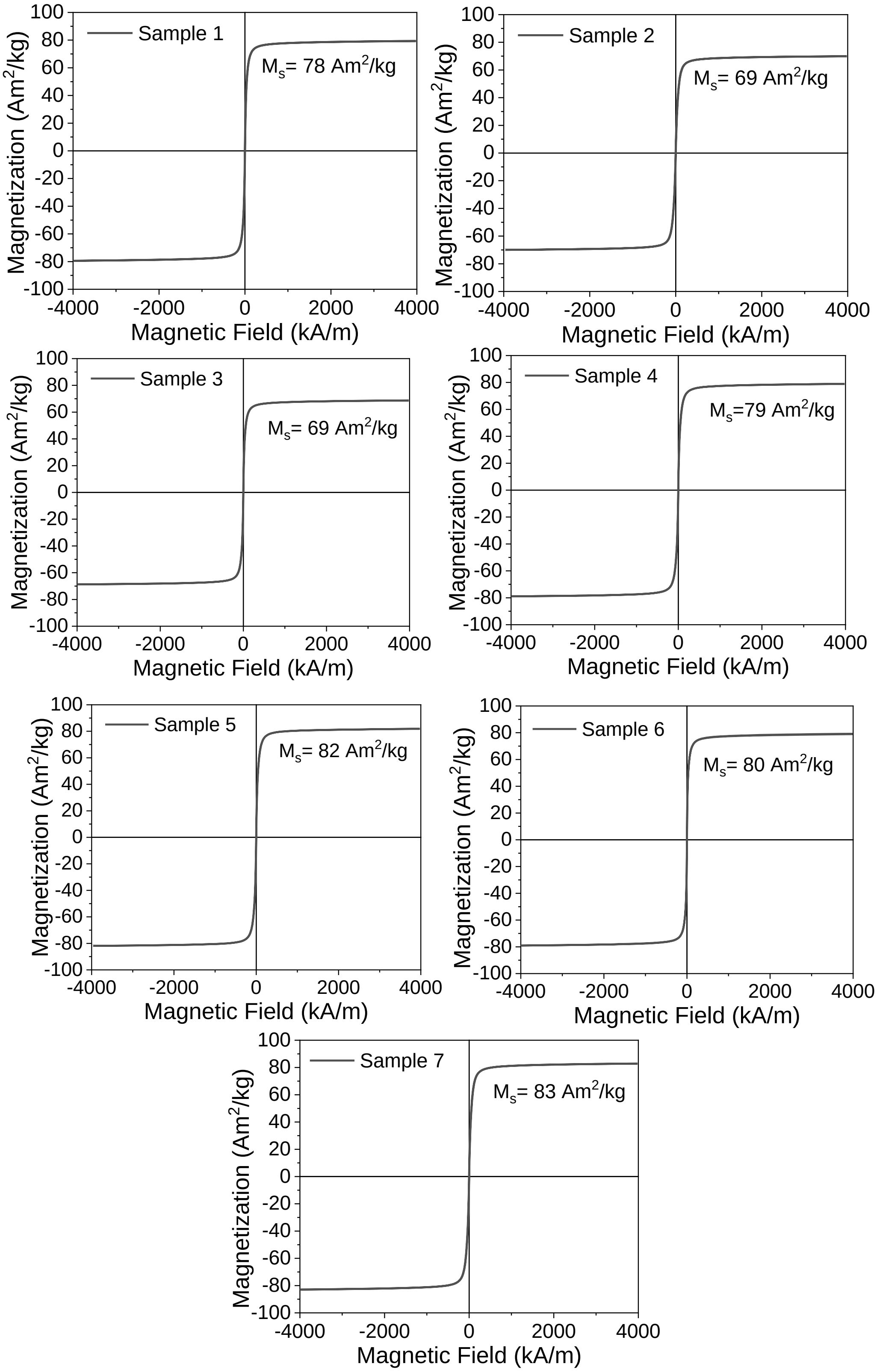

Figure S11. Hysteresis loops at 290 K of NF4 batches synthesized automatically by an arm robot and carrousel within the Anton Paar® Monowave Microwave to check their reproducibility.

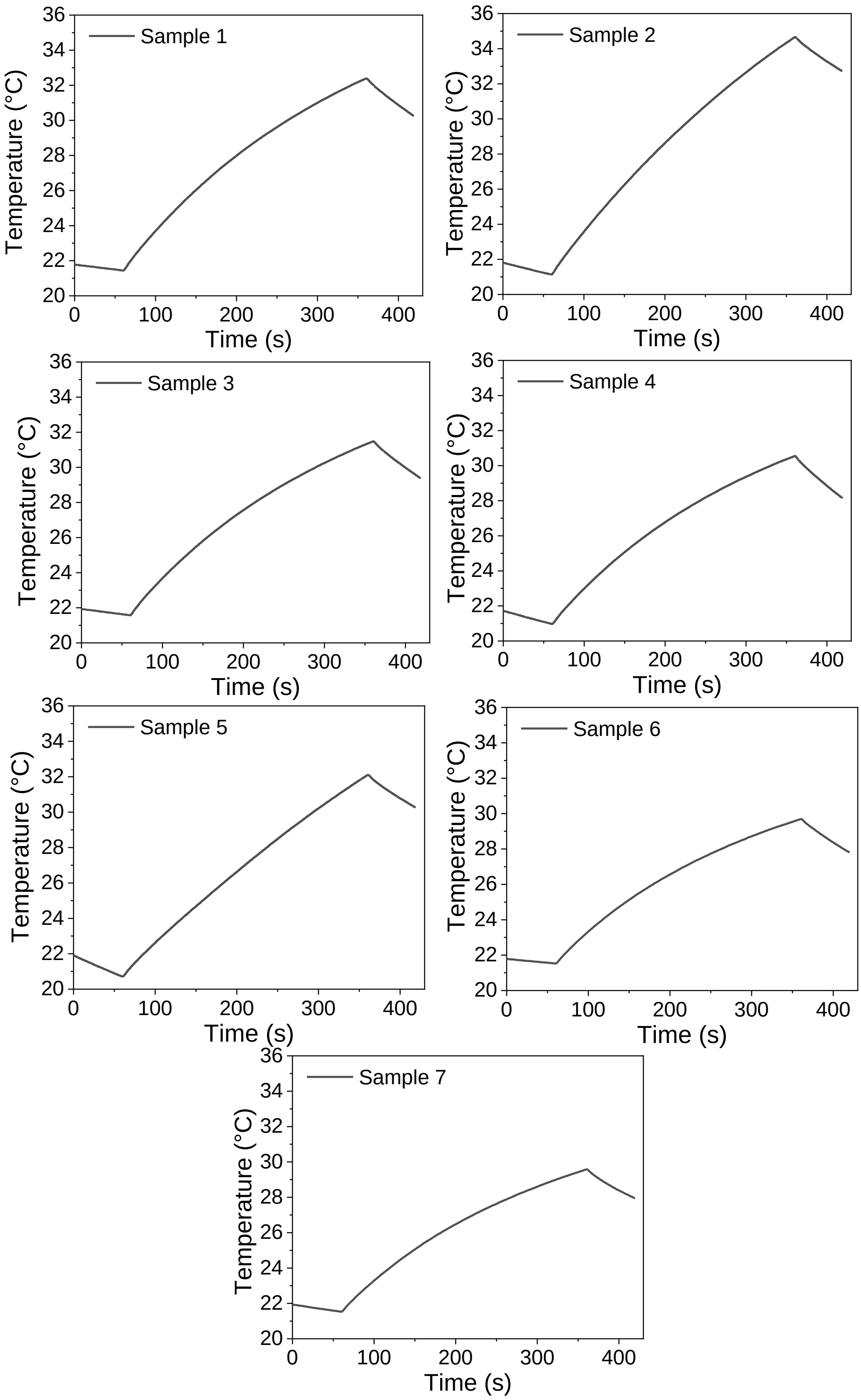

Figure S12. Heating graphs of NF4 batches in the function of time using an alternating magnetic field of 24 kA m^{-1} and a frequency of 200 kHz.

Table S1. Structural and colloidal parameters of the NF4 batches synthesized by the microwave-assisted method

Samples	Particle size	Core size	Crystal size	Coercitivity*	χ	Magnetization at 290 K	SAR 200 kHz, 24 kA m^{-1}	Yield
	(nm)	(nm)	(nm)	(kA m^{-1})		(Am ² kg^{-1})	(W g_{NPS}^{-1})	(mg)
Sample 1	39 ± 7	7 ± 2	15.4	2.1	1.4	78.1	192.5	27.2
Sample 2	40 ± 6	6 ± 1	13.8	1.8	1.2	68.8	203.6	34
Sample 3	41 ±6	6 ± 2	14	2.1	1.2	69.2	182.2	35.6
Sample 4	40 ± 6	6 ± 2	14.5	1.7	1.2	79.5	173.5	36.8
Sample 5	41 ± 6	7 ± 1	13.7	1.8	1.3	81.8	164	30.4
Sample 6	43 ± 8	6 ± 1	13.9	1.5	1.6	79.8	152.2	36.1
Sample 7	44 ± 6	7 ± 2	14	1.7	1.1	83.5	149.1	37.1

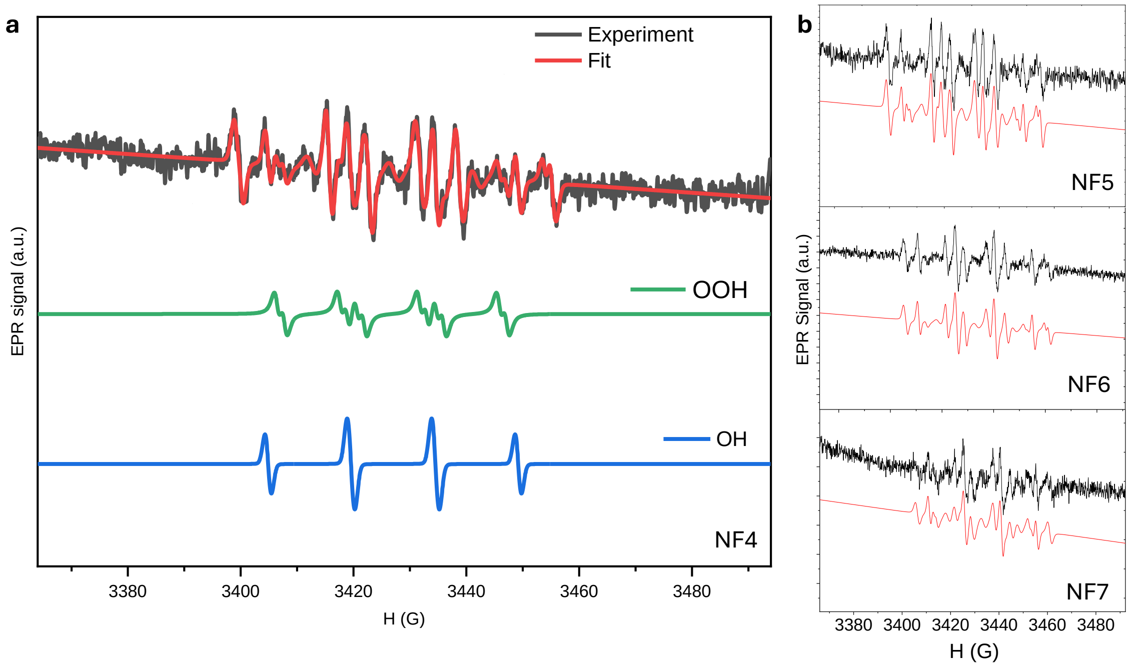

Figure S13. a. EPR Spectra of sample NF4 and deconvolution of •OOH and •OH radicals. **b.** EPR Spectra of samples NF5, NF6 and NF7.

Table S2. Maximum area of free radicals generated by iron oxide nanoflowers (a.u.).

Sample	•OOH	•OH
NF4	0.134	0.081
NF5	0.270	0.087
NF6	0.321	0.210
NF7	0.641	0.147

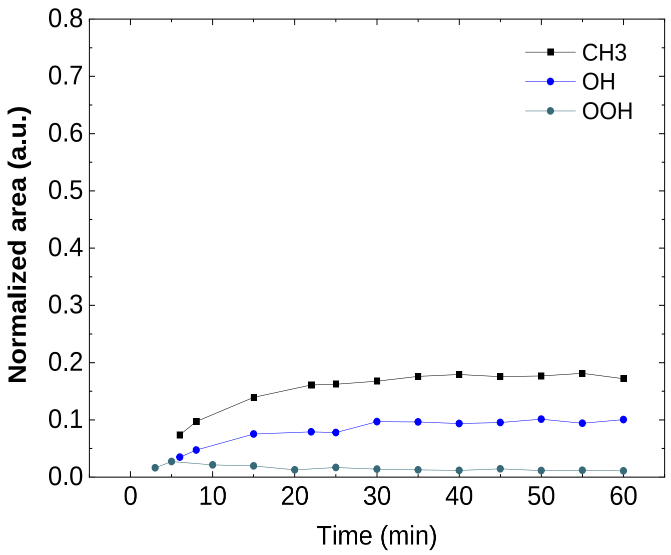

Figure S14 Control experiment for ROS detection monitored by EPR spectroscopy in the absence of nanoflowers (blank). No significant signal corresponding to hydroperoxyl (•OOH) or hydroxyl (•OH) radicals is observed, confirming that radical generation arises from the presence of the nanoflowers.
